# Polarisierung oder Verringerung ungleicher Teilnahmechancen? Auswirkungen der COVID-19-Pandemie auf das berufliche Lernen im Erwachsenenalter

**DOI:** 10.1007/s11618-023-01142-2

**Published:** 2023-02-21

**Authors:** Corinna Kleinert, Gundula Zoch

**Affiliations:** 1 Leibniz-Institut für Bildungsverläufe, Otto-Friedrich-Universität Bamberg, Wilhelmsplatz 3, 96047 Bamberg, Deutschland; 2 Leibniz-Institut für Bildungsverläufe, Carl von Ossietzky Universität Oldenburg, Ammerländer Heerstraße 114–118, 26129 Oldenburg, Deutschland

**Keywords:** Weiterbildung, Berufliche Weiterbildung, Nonformales Lernen, Informelles Lernen, Covid-19, Corona, Pandemie, Soziale Ungleichheit, Matthäus-Effekte, Adult education, Job-related further training, Nonformal learning, Informal learning, Covid-19, Pandemic, Social inequality, Matthew effects

## Abstract

In politischen und wissenschaftlichen Diskursen wird immer wieder betont, wie wichtig das lebenslange Lernen in den heutigen Wissensgesellschaften ist. Gleichzeitig ist die Teilnahme an berufsbezogener Weiterbildung (BWB) aber nach wie vor sozial stratifiziert: Es profitieren vor allem diejenigen, die sowieso schon bessere Voraussetzungen und mehr Ressourcen mitbringen. Obgleich die Corona-Pandemie das Angebot an und die Nachfrage nach Weiterbildung in kurzer Zeit tiefgreifend verändert hat, ist insgesamt noch nicht abzusehen – und empirisch bislang nicht ausreichend untersucht – wie sich die Pandemie auf die Beteiligung an BWB ausgewirkt hat und welche Barrieren und Chancen die Krise für unterschiedliche Beschäftigtengruppen mit sich gebracht hat. Diese Fragen beantworten wir empirisch auf Basis der Daten der NEPS-Startkohorte 6 bei einer Stichprobe Erwachsener, die vor und während der Corona-Krise an den NEPS-Befragungen teilgenommen hat und kurz vor dem Ausbruch der Pandemie erwerbstätig war. Unsere Ergebnisse zeigen, dass die Covid-19-Pandemie in Deutschland mit einem moderaten Rückgang der Teilnahme an berufsbezogenen Kursen und Präsenzveranstaltungen einherging. Die vor der Pandemie stark ausgeprägten sozialen, beruflichen und betrieblichen Differenzen in diesen Beteiligungsformen sind im Zuge der Pandemie ebenfalls leicht zurückgegangen. Wir schließen daraus, dass die Pandemie zumindest in ihrer ersten und zweiten Welle zu einem Rückgang der sozialen Ungleichheit von Weiterbildung geführt hat.

**Zusatzmaterial online:**

Zusätzliche Informationen sind in der Online-Version dieses Artikels (10.1007/s11618-023-01142-2) enthalten.

## Abstract (en)

Political and scientific discourses repeatedly emphasize the importance of lifelong learning in today’s knowledge societies. At the same time, participation in vocational further education (VFE) is still socially stratified: mainly those adults profit who already bring with them better qualifications and more resources. The Corona pandemic has profoundly changed the supply with and the demand for further education in a short period of time, and it is not yet clear—and empirically not sufficiently investigated—how the pandemic has affected participation in VFE and what barriers and opportunities the crisis has brought for different groups of employees. We answer these questions empirically using data from NEPS Start Cohort 6 for a sample of adults, who participated in the NEPS surveys before and during the Corona crisis and were employed shortly before the outbreak of the pandemic. Our results show that the Covid-19 pandemic in Germany was associated with a moderate decline in participation in job-related courses and face-to-face events. The social, occupational, and workplace differences in these forms of participation, which were pronounced before the pandemic, also declined slightly in the wake of the crisis. We conclude that the pandemic has led to a decrease of social inequality of adult education, at least in its first and second wave.

## Einleitung

Die Covid-19-Pandemie prägt den Alltag der Menschen weltweit seit über zwei Jahren und hat viele Bereiche davon maßgeblich verändert. Besonders intensiv diskutiert und beforscht werden potenziell negative Auswirkungen von Maßnahmen zur Eindämmung der Pandemie im Bereich der formalen – und hier vor allem der schulischen und hochschulischen – Bildung. Andere Formen von Bildung stehen bisher deutlich weniger im Fokus bildungspolitischer Diskurse und medialer Aufmerksamkeit. Das gilt in besonderem Maße für die berufsbezogene Weiterbildung (BWB), vermutlich, weil diese Form des Lernens nicht institutionalisiert erfolgt,^1^ die Lernphasen kürzer sind und in vielfältigen Kontexten stattfinden. Folglich lässt sich die Beteiligung daran auch nicht so einfach erfassen wie im Bereich formaler Bildung (Ehlert et al. 2020).

Die Pandemie hat jedoch sowohl das Angebot an dieser Form des Lernens als auch die Nachfrage nach ihr in kurzer Zeit tiefgreifend verändert (Ehlert et al. 2020). Einerseits ist die traditionelle berufsbezogene Weiterbildung in Form von Präsenzkursen weitgehend zusammengebrochen (Bundesinstitut für Berufsbildung 2021; Wuppertaler Kreis 2020), und etliche Betriebe haben ihre Weiterbildungsinvestitionen aufgrund von Maßnahmen des Infektionsschutzes sowie ökonomischer Unsicherheit reduziert (Bellmann et al. 2020; Leifels 2021). Andererseits hat die Krise einen Digitalisierungsschub ausgelöst, der es für viele Beschäftigte notwendig gemacht hat, in kurzer Zeit neue Dinge zu erlernen (Euler und Severing 2020; Seyda 2021). Im Rahmen dieser Entwicklung entstanden auch neue digitale Bildungsangebote (Flake et al. 2020). Gleichzeitig sind bei einigen Beschäftigtengruppen, bedingt durch Kurzarbeit und das Arbeiten von zuhause aus, neue zeitliche Spielräume für berufsbezogene Weiterbildung entstanden, während andere aufgrund von Mehrarbeit und geschlossener Kinderbetreuungseinrichtungen und Schulen weniger Zeit dafür hatten als zuvor.

Diese kursorischen Überlegungen deuten bereits an, dass die Auswirkungen der Pandemie auf die Beteiligung an berufsbezogener Weiterbildung vermutlich komplex sind. Insgesamt ist noch nicht abzusehen – und empirisch bislang noch kaum untersucht – welche Teilnahmechancen und -barrieren die Krise für unterschiedliche Beschäftigtengruppen mit sich gebracht hat (Denninger und Käpplinger 2021). Von besonderer Bedeutung ist dabei die Frage, ob die Krise zu einem Rückgang der sozialen Ungleichheit bei der Beteiligung an berufsbezogener Weiterbildung geführt hat, die grundsätzlich von starken sozialen Ungleichheiten nach Bildung und Qualifikationsniveau geprägt ist (Kilpi-Jakonen et al. 2015), oder ob sich diese Ungleichheiten umgekehrt verstärkt haben.

Diese Fragen beantworten wir für Deutschland empirisch auf Basis der Längsschnittdaten der Startkohorte 6 des Nationalen Bildungspanels (NEPS), für die unterschiedliche Lernformen für eine große Stichprobe Erwachsener mit heterogenen Voraussetzungen und Erwerbsverläufen langjährig wiederholt erfasst worden sind. Wir beschreiben zunächst allgemeine theoretische und empirische Überlegungen zu den nachfrage- und angebotsseitigen Faktoren für bestehende soziale Ungleichheiten in der Teilnahme an nonformaler und informeller BWB. Im Anschluss daran spezifizieren wir diese Überlegungen zu Ausmaß und Form krisenbedingter Veränderungen und untersuchen empirisch, wie die Pandemie die Weiterbildungsbeteiligung erwerbstätiger Erwachsener beeinflusst hat und welche Rolle dabei individuelle Voraussetzungen und betriebliche Opportunitäten gespielt haben.

## Theoretische Überlegungen und Forschungsbefunde

In Deutschland bilden sich Erwachsene nach ihrem Einstieg ins Erwerbsleben nur selten formal durch den Erwerb voll qualifizierender schulischer, beruflicher oder tertiärer Abschlüsse weiter (etwa indem sie das Abitur nachholen, ein Studium absolvieren oder die Meisterschule besuchen). Das Gros des Lernens im Erwachsenenalter erfolgt entweder in nonformalen oder informellen Kontexten. *Nonformale Bildung *findet außerhalb des institutionalisierten Bildungssystems in Form von kürzeren organisierten Aktivitäten wie Kursen oder Schulungen statt. Diese können, müssen jedoch nicht mit einem Zertifikat abgeschlossen werden. *Informelles Lernen *ist selbstgesteuert, nicht organisiert und vollzieht sich durch tägliche Erfahrungen, beispielsweise in der Freizeit, im Familienkreis oder am Arbeitsplatz (Tuijnman und Boström 2002). Ausgehend von dem Befund, dass gerade die Rolle des informellen Lernens in der Pandemie wenig untersucht wurde (Denninger und Käpplinger 2021), konzentrieren wir uns im Folgenden auf nonformales und informelles Lernen von Erwachsenen.^2^ Wir beziehen uns dabei nur auf *berufsbezogene* Lernaktivitäten, weil diese für den Erhalt und die Entwicklung von Kompetenzen, die auf dem Arbeitsmarkt benötigt werden, besonders relevant sind und in besonderer Weise von der Krise betroffen waren. Diese können sowohl vom Arbeitgeber als auch von den Teilnehmenden selbst organisiert und finanziert sein sowie innerhalb oder außerhalb der Arbeitszeit erfolgen.

### Soziale Ungleichheiten in der Beteiligung an berufsbezogener Weiterbildung

In politischen ebenso wie in wissenschaftlichen Diskursen wird immer wieder betont, wie wichtig das lebenslange Lernen in den heutigen Wissensgesellschaften ist (van Nieuwenhove und de Wever 2021). Der technologische Wandel und die schnell voranschreitende Digitalisierung erfordern auf vielen Arbeitsplätzen eine kontinuierliche Aneignung neuer Fertigkeiten und Technologien, die nur durch einen Ausbau berufsbezogener Weiterbildung zu bewältigen ist (Euler und Severing 2020). In Deutschland kommt dem lebenslangen berufsbezogenen Lernen vor dem Hintergrund der demografischen Alterung der Erwerbsbevölkerung eine besonders starke Bedeutung zu, denn neue Arbeitsroutinen und Technologien können in Betrieben nicht einfach dadurch implementiert werden, dass junge, spezialisierte Fachkräfte neu eingestellt werden (Becker 2018). Entsprechend zeigen die Zahlen des Adult Education Survey (AES), der das Weiterbildungsverhalten in Deutschland alle zwei Jahre erfasst, einen kontinuierlichen Anstieg der Weiterbildungsbeteiligung seit 2010. Im Jahr 2020 beteiligten sich insgesamt 60 % der 18- bis 64-jährigen Bevölkerung an Weiterbildungen; über 80 % davon aus beruflichen Gründen (BMBF 2021).^3^ Darüber hinaus gaben 46 % an, informell mit Büchern oder Fachzeitschriften gelernt zu haben, und 41 % nutzten computer- oder internetbasierte Lernangebote (BMBF 2021).^4^

Gleichzeitig ist die Teilnahme an berufsbezogener Weiterbildung sozial stratifiziert, das heißt von sogenannten *Matthäus-Effekten *gekennzeichnet: Es partizipieren vor allem diejenigen, die sowieso schon bessere Voraussetzungen mitbringen (Kilpi-Jakonen et al. 2015). Je höher Beschäftigte gebildet sind und je qualifizierter und anspruchsvoller ihre Arbeitsaufgaben sind, desto eher investieren ihre Arbeitgeber in Weiterbildung und desto wahrscheinlicher entscheiden sie sich auch selbst dafür, kontinuierlich weiterzulernen (Kleinert und Wölfel 2018). Entgegen der landläufigen Annahme, dass informelles Lernen niedrigschwelliger sei als organisierte Lernformen, gibt es auch hier ausgeprägte Matthäus-Effekte, insbesondere im Bereich des medialen Lernens (Kaufmann 2016; Kuwan und Seidel 2013). Zudem korrelieren die Beteiligung an nonformaler und die an informeller Bildung positiv miteinander (Kaufmann 2016), so dass ähnliche Mechanismen für die sozialen Ungleichheiten in der Beteiligung an nonformalem und informellem berufsbezogenem Lernen zu vermuten sind.

Um die sozialen Ungleichheiten in der Beteiligung an berufsbezogener Weiterbildung und die pandemiebedingten Veränderungen im Folgenden zu erklären, nehmen wir einerseits die individuellen Voraussetzungen der Beschäftigten in den Blick, welche die Nachfrage nach Weiterbildung bestimmen, und andererseits die angebotsseitigen Gelegenheitsstrukturen, die eine Beteiligung ermöglichen oder erschweren (vgl. dazu näher Kaufmann und Widany 2013; Schiener 2006).^5^ Theoretisch knüpfen wir dabei vorrangig an das Wert-Erwartungs-Modell, rationale Entscheidungstheorien, humankapitaltheoretische Überlegungen und Ansätze der Arbeitsmarktsegmentation an. Wir beschränken uns dabei auf Ansätze, die einen Bezug zu sozialen Ungleichheiten in der Weiterbildungsbeteiligung und deren Veränderungen in der Pandemie erwarten lassen.

*Nachfrageseitige* Faktoren sind für die Erklärung von sozialen Ungleichheiten in der Weiterbildungsbeteiligung immer dann von besonders großer Bedeutung, wenn es um Bildungsaktivitäten geht, die nicht betrieblich initiiert sind, sondern von den Lernenden selbst organisiert und gesteuert werden (Kaufmann und Widany 2013). Um sich für berufsbezogenes Lernen zu entscheiden, sind zunächst motivationale Faktoren, aber auch Anforderungen an kognitive Lernvoraussetzungen (Kaufmann 2016) bedeutsam, insbesondere bei informellen Lernprozessen. Diese Zusammenhänge können sehr gut mit dem Erwartungs-Wert-Modell von Eccles (2005) sowie Wigfield und Eccles (2000) erklärt werden (Gorges 2015). Danach beeinflusst das sozio-kulturelle Umfeld die Lernerfahrungen einer Person in der Kindheit und Jugend, und beide wirken sich auf das Selbstkonzept einer Person aus, das wiederum die Erfolgserwartung bestimmt. Frühere Lernerfahrungen beeinflussen darüber hinaus den subjektiven Wert, der einer Weiterbildung zugemessen wird. Sowohl Erfolgserwartungen als auch Wertüberzeugungen sind schließlich Voraussetzungen für eine Teilnahme an Weiterbildungsangeboten (für eine genauere Diskussion vgl. Gorges 2015). Über dieses Modell lassen sich folglich Beteiligungsunterschiede zwischen niedrig und hoch gebildeten bzw. qualifizierten Erwachsenen insbesondere an selbst organisierten beruflichen Lernaktivitäten gut erklären. Dieser theoretische Rahmen legt außerdem nahe, dass Wertüberzeugungen zu Weiterbildung nicht nur durch individuelle Erfahrungen, sondern auch sozialstrukturell und kulturell geprägt sind. Sie können daher nicht nur bei verschiedenen Bildungs- und Berufsgruppen, sondern auch in West- und Ostdeutschland sowie bei Personen mit und ohne Migrationshintergrund systematisch anders ausfallen. Bei Untersuchung der Weiterbildungsbeteiligung letzterer Gruppe ist außerdem zu berücksichtigen, dass ausländische Bildungsabschlüsse in Deutschland oft nicht anerkannt werden und gegebenenfalls sprachliche Hürden sowie eine Bleibeunsicherheit eine Weiterbildungsteilnahme erschweren (Reiter 2022).

Über motivationale Differenzen hinaus erklären Theorien des rationalen Investitionsverhaltens (Becker 2018), warum höher gebildete Erwachsene und solche in statushöheren Berufen eher weiterlernen als andere: Sie sind motiviert, ihre durch frühere Investitionen erworbene hohe Bildung auf dem neuesten Stand zu halten, erwarten (zu Recht) höhere Erfolgschancen und höhere künftige Erträge und sind in der Regel besser über Lernmöglichkeiten informiert als andere (Beicht und Walden 2006). Außerdem haben sie geringere direkte und indirekte Kosten zu tragen als geringer Gebildete (Becker 2018); dies gilt vor allem für direkte monetäre Kosten und Opportunitätskosten des Lernens. Aber auch zeitliche Kosten dürften für höher Gebildete geringer ausfallen, da diese im Vergleich zu geringer Gebildeten mehr finanzielle Mittel haben, um häusliche Arbeiten, Pflege und Erziehung an Dritte abzugeben. Da die erwarteten Gesamterträge berufsbezogener Weiterbildung über die Lebensspanne geringer ausfallen, wenn Beschäftigte nach der Teilnahme weniger Zeit in Erwerbstätigkeit verbringen, nimmt die Beteiligung mit dem Lebensalter ab (Becker 2018). Ebenso benennen Frauen geringere zeitliche und finanzielle Spielräume (und damit höhere subjektive Kosten) für eine Weiterbildungsteilnahme als Männer, da sie in der Regel die Hauptlast der familiären Haus‑, Pflege- und Erziehungsarbeit tragen, ihre Verdienste und Karriereaussichten oftmals geringer sind und sie seltener von betrieblicher Unterstützung bei der Weiterbildung profitieren können (Dieckhoff und Steiber 2011; Zoch 2022).

Auf der *Angebotsseite* spielen Betriebe eine besondere Rolle für die Ermöglichung von berufsbezogenem Lernen. Dies lässt sich mit Zahlen des AES verdeutlichen: So waren in Deutschland im Jahr 2018 72 % aller privaten und beruflichen Weiterbildungsaktivitäten der 18- bis 64-jährigen Bevölkerung betriebliche Weiterbildungen und weitere zehn Prozent individuelle berufsbezogene Weiterbildungen (BMBF 2021). Der berufliche Weiterbildungsmarkt ist in Deutschland überwiegend privat organisiert. Die Angebote öffentlicher Träger wie die der Volkshochschulen stehen zwar prinzipiell der gesamten Bevölkerung offen, zielen aber nicht vorrangig auf die Vermittlung berufsbezogenen Wissens ab. Neben betrieblichen Weiterbildungen finden sich insbesondere im Bereich digitaler Kompetenzen und IT-Zertifikate zahlreiche nonformale Bildungsangebote privater Anbieter, die entweder von den Lernenden selbst oder deren Betrieben finanziert werden müssen (Euler und Severing 2020).

Nach humankapitaltheoretischen Annahmen bieten Betriebe Weiterbildung allerdings nur dann an oder kaufen diese ein, wenn es ihnen sinnvoll erscheint, in die Produktivität ihrer Mitarbeiter zu investieren – zum Beispiel um die Abwanderung von Fachkräften zu vermeiden, um ihre eigene Position auf wettbewerblichen Märkten zu verbessern oder um sehr produktive Mitarbeiter aus der Masse der Beschäftigten herauszufiltern (Becker 2018). Aus diesem Grunde nimmt das Angebot mit Anstieg des Qualifikationsniveaus der Beschäftigten und ihrer Arbeitszeit zu, mit Zunahme ihres Alters und der Dauer ihrer Betriebszugehörigkeit hingegen ab (Kaufmann und Widany 2013).

Ergänzend dazu legen segmentationstheoretische Annahmen nahe, dass die Weiterbildungsmöglichkeiten auf betriebsinternen und berufsspezifischen Arbeitsmärkten, wo qualifizierte Arbeitskräfte weitgehend vom Marktwettbewerb abgeschirmt beschäftigt sind, höher sind als auf Jedermanns-Arbeitsmärkten (Schiener et al. 2013). Daher investieren beispielweise größere Betriebe deutlich mehr in die Weiterbildung ihrer Mitarbeiter als kleinere und verfügen in der Folge häufiger über eigene Weiterbildungsprogramme, -zuständige und Betriebsvereinbarungen über Weiterbildung. Darüber hinaus hängen die betrieblichen Strukturen und die Finanzierung von Weiterbildung eng mit dem Verhältnis von Arbeitsplätzen und Technologie zusammen. Der Weiterbildungsbedarf ist am höchsten auf technologieergänzenden Arbeitsplätzen, auf denen vorrangig Wissensarbeit stattfindet, geringer auf technologiebegleitenden Arbeitsplätzen und am geringsten auf Arbeitsplätzen, die durch Technologie ersetzbar sind. Aus diesen Gründen erhöht der technologische Wandel den betrieblichen Bedarf nach beruflicher Weiterbildung, insbesondere auf den internen Arbeitsmärkten, und verstärkt somit die Segmentierung der Arbeitsmärkte (Becker 2018).

### Auswirkungen der Pandemie auf berufsorientierte Weiterbildung

Die Corona-Pandemie und die damit verbundenen Maßnahmen des Infektionsschutzes haben das Angebot von als auch die Nachfrage nach berufsbezogener Weiterbildung in Deutschland in kurzer Zeit tiefgreifend verändert. Dennoch gibt es bisher nur wenig verlässliche und uneinheitliche Evidenz dazu, wie sich die *Teilnahme* an berufsbezogener Weiterbildung seit dem Beginn der Pandemie entwickelt hat. Nach Zahlen des Adult Education Surveys 2020 hat die BWB gegenüber der Vorwelle im Jahr 2018 nicht etwa abgenommen, sondern ist deutlich angestiegen (BMBF 2021). Gleichzeitig ging die Dauer der Bildungsaktivitäten beträchtlich zurück, insbesondere im Feld der individuellen beruflichen Weiterbildung (BMBF 2021). Da ein erheblicher Teil des Erfassungszeitraums für Bildungsaktivitäten im AES vor dem Beginn der Krise lag und sowohl die Stichprobenziehung als auch der Modus der Befragung gegenüber der Vorwelle verändert wurden, sind beide Messzeitpunkte jedoch nur bedingt miteinander vergleichbar. Verlässliche Aussagen über Veränderungen im Zuge der Pandemie lassen sich daraus kaum ableiten. Im Vergleich dazu kommen Simulationen (OECD 2021) und Betriebsbefragungen (International Labour Organization 2021; Jost und Leber 2021) sowohl international als auch für Deutschland zu deutlich pessimistischeren Einschätzungen.

#### Das veränderte Weiterbildungsangebot

Die bisher uneinheitliche Evidenz zur Entwicklung der berufsbezogenen Weiterbildungsbeteiligung ergibt sich in Teilen aus den unterschiedlichen und teilweise gegensätzlichen Auswirkungen der Corona-Pandemie auf die Angebots- und Nachfrageseite von BWB. Einerseits veränderte sich krisenbedingt das *Angebot* an traditionellen Präsenzveranstaltungen, die betrieblich organisiert sind oder von öffentlichen und privaten Anbietern durchgeführt werden. Diese Angebotsformen brachen in den Phasen mit rigiden Maßnahmen zur Kontaktreduzierung weitgehend zusammen (Bundesinstitut für Berufsbildung 2021; Wuppertaler Kreis 2020), und in den Phasen gelockerter Kontaktbeschränkungen waren sie aufgrund der weiterhin geltenden Abstandsregelungen und der mangelnden Teilnahmebereitschaft ökonomisch wenig ertragreich (Schmidt-Hertha 2021). So mussten in Deutschland viele Betriebe geplante Weiterbildungsveranstaltungen krisenbedingt wieder absagen, und nur zehn Prozent der Betriebe nutzten Zeiten des Arbeitsausfalls in der Krise für Weiterbildung (Bellmann et al. 2021). Darüber hinaus verringerten viele Betriebe in der Privatwirtschaft angesichts pandemiebedingter Schließungen, unterbrochener Geschäftstätigkeiten und der wirtschaftlichen Unsicherheit im Zuge der Krise bewusst ihre Weiterbildungsinvestitionen (Bellmann et al. 2020; Leifels 2021).

Andererseits beschleunigte die Krise bereits vorher bestehende Digitalisierungstendenzen rapide und führte in der Folge zu erhöhten Weiterbildungsbedarfen der Betriebe (Seyda 2021): Arbeitsprozesse wurden, wo möglich, digital kompatibel gemacht und ins Homeoffice verlagert, und die dafür notwendige Infrastruktur wurde sehr schnell weiterentwickelt und nachgesteuert (Euler und Severing 2020). Entsprechend mussten Beschäftigte neue digitale Kompetenzen erlernen oder vorhandenes Wissen auffrischen. Daher nahm – anders als bei Präsenzveranstaltungen – die Beteiligung an informellen, insbesondere an digitalen Lernformen nach übereinstimmenden Berichten zu (BMBF 2021; Kleinert et al. 2021b), wobei digitale Lernaktivitäten häufiger weniger intensiv ausfallen als das Lernen vor Ort (BMBF 2021). Insgesamt ist zu erwarten, dass (informelle) digitale Lernangebote während der Pandemie erheblich zugenommen haben (Flake et al. 2020).

Es ist allerdings nicht zu erwarten, dass diese dargestellten Trends einheitlich und in gleicher Weise bei unterschiedlichen Beschäftigtengruppen aufgetreten sind (für erste internationale Evidenz dazu vgl. Li et al. 2020). Betriebliche Investitionskalküle mit Blick auf Produktivitätsannahmen anhand des beruflichen Status, der Arbeitszeit, der Betriebszugehörigkeit oder des Alters der Beschäftigten dürften sich durch die Pandemie erst einmal nicht grundsätzlich verändert haben. Die Pandemie führte allerdings insbesondere zu ihrem Beginn dazu, dass sich betrieblich induzierte Bedarfe für Weiterbildungen insgesamt abrupt wandelten. In vielen Betrieben dürften die unvermittelten organisatorischen Veränderungen besonders in der Anfangsphase der Corona-Krise zu einem neuen Qualifizierungs- und Weiterbildungsbedarf geführt haben (Kleinert et al. 2021a). Dabei ist allerdings anzunehmen, dass nicht alle Betriebe genügend Ressourcen hatten, um (Online‑)Schulungen für ihre Beschäftigten zu organisieren. Größeren und technologienahen Betrieben mit einer etablierten Weiterbildungsstruktur dürfte das leichter gefallen sein als kleineren und technologieferneren Unternehmen ohne diese organisationalen Ressourcen (Leifels 2021; Seyda 2021).

Generell ist anzunehmen, dass sich betriebliche und individuelle Investitionskalküle in Abhängigkeit von der Betroffenheit durch die Krise verändert haben, d. h. je nachdem, ob und wie Kosten- und Nutzenerwartung von Weiterbildung bei Betrieben und Weiterbildungsanbietern durch die Auswirkungen der Pandemie beeinflusst wurden. Diese Veränderungen unterscheiden sich zum einen nach den Spielräumen, welche während der Krise für Weiterbildung zur Verfügung standen, und zum anderen nach den Notwendigkeiten für berufsbezogenes Lernen aufgrund der Krise. Dabei sind drei spezifische Beschäftigungsbedingungen zu berücksichtigen, die in der Pandemie besondere Bedeutung erlangt haben: das Arbeiten von zuhause aus, die Beschäftigung in systemrelevanten Berufen sowie das Instrument der Kurzarbeit.

Ab Beginn der Kontaktbeschränkungen, spätestens jedoch mit der gesetzlichen Homeoffice-Pflicht für Arbeitgeber ab Januar 2021, arbeiteten große Teile der Beschäftigten ganz oder zumindest teilweise im *Homeoffice*. So war ein beträchtlicher Teil der Beschäftigten gezwungen, schnell neue Fähigkeiten zu erlernen oder aufzufrischen, um von zu Hause aus mit neuen kollaborativen digitalen Werkzeugen arbeiten zu können (Kleinert et al. 2021b). Solche Fähigkeiten dürften oftmals informell und digital erlernt bzw. vermittelt worden sein. Erste Ergebnisse zeigen, dass höher qualifizierte Beschäftigte mit Zugang zum Homeoffice vom Online-Lernen deutlich stärker profitieren konnten als niedriger qualifizierte Beschäftigte und solche, die während der Krise vor Ort arbeiten mussten (Kleinert et al. 2021b). Umgekehrt gibt es jedoch auch Hinweise darauf, dass sich das digitale Lernen während der Krise auf basale und kurzfristig erlernbare Digitalkompetenzen fokussierte (Leifels 2021), von denen wiederum ggf. gerade niedriger qualifizierte Beschäftigte profitiert haben könnten.

Jenseits dessen mussten auch viele Beschäftigte, die weiter in Betrieben vor Ort arbeiteten, mit organisatorischen Veränderungen im Arbeitsalltag umgehen, zum Beispiel mit neuen Hygienevorschriften oder veränderten Arbeitsabläufen, und dazu neue Dinge erlernen. Gerade in *systemrelevanten Berufen* (Koebe et al. 2020) dürfte der akute Weiterbildungsbedarf zumindest zu Beginn der Pandemie sehr hoch gewesen sein, weil neue Regelungen am Arbeitsplatz beachtet werden mussten und sich viele Routinen veränderten, beispielsweise im Einzelhandel, in Krankenhäusern oder in Schulen. Andererseits dürfte es in vielen dieser Bereiche kaum größere zeitliche Spielräume für Bildungsaktivitäten gegeben haben, weil die Veränderungen sehr schnell umgesetzt werden mussten und oft keine ausreichenden Personalressourcen zur Verfügung standen, um Lernzeiten abzupuffern.

Hinsichtlich der Opportunitäten der Betriebe selbst ist anzunehmen, dass vor allem Wirtschaftszweige, die von der Pandemie besonders betroffen waren, ihre Investitionen in die Weiterbildung ihrer Beschäftigten seit Ausbruch der Pandemie verringert haben. Dies betrifft zum Beispiel das verarbeitende Gewerbe, den Tourismus oder das Gastgewerbe. Da dies Branchen mit einer insgesamt eher gering qualifizierten Beschäftigtenstruktur sind, spricht dies – unter der zuvor genannten Annahme – dafür, dass sich soziale Ungleichheiten in der Weiterbildungsbeteiligung in der Krise weiter verstärkt haben. In Deutschland spielte für diese Betriebe das Instrument des *Kurzarbeitergeldes* eine wesentliche Rolle. Dadurch wurde gewährleistet, dass Beschäftigte trotz verminderter Produktion oder vollständiger Werksschließungen nicht entlassen werden mussten. Dennoch dürften betroffene Betriebe oftmals keine finanziellen Spielräume gehabt haben, um in dieser Zeit systematisch in die Weiterbildung ihrer Beschäftigten zu investieren.

#### Die veränderte Nachfrage nach Weiterbildung

Auf der Nachfrageseite hat die Pandemie unserer Einschätzung nach insgesamt weniger Veränderungen erzeugt als auf der Angebotsseite der Betriebe. Viele individuelle Voraussetzungen der Beschäftigten für das Lernen sind von der Krise zumindest in kürzerer Sicht unberührt geblieben, z. B. kognitive Voraussetzungen, Bildungserfahrungen, Erfolgserwartungen oder der Wert, den sie Bildung zumessen. Veränderungen haben sich hingegen bei zeitlichen, monetären und motivationalen Kosten ergeben. So gehen wir davon aus, dass die Entscheidung, in Weiterbildung zu investieren, durch zeitliche Einschränkungen aufgrund von Familienarbeit und ‚home schooling‘, denen erwerbstätige Mütter mit kleinen oder schulpflichtigen Kindern besonders stark ausgesetzt waren (Zoch et al. 2021), negativ beeinflusst worden ist. Auch eine größere Arbeitsbelastung im Beruf, z. B. in systemrelevanten Berufen im Gesundheitsbereich oder in Branchen, die von der Krise wenig betroffen waren und die ihre Arbeitsabläufe schnell auf das Arbeiten von zuhause aus umstellen konnten, könnte solche zeitlichen Restriktionen mit sich gebracht haben.

Anderen Beschäftigtengruppen eröffnete die Krise dagegen neue zeitliche Spielräume für Weiterbildung, etwa weil durch wegfallende Pendelzeiten Zeitersparnisse für selbst organisiertes Lernen außerhalb der Arbeitszeit entstanden sind oder weil sich ihr Arbeitsvolumen durch Werks- bzw. Büroschließungen oder unterbrochener Geschäftstätigkeit verringert hat. Letzteres gilt insbesondere für Beschäftigte in Kurzarbeit. Umgekehrt musste diese Gruppe aber mit Einkommensverlusten umgehen; vor allem aber hat Kurzarbeit die Besorgnis über zukünftige Unsicherheiten verstärkt (Frodermann et al. 2020), was sich wiederum hemmend auf die Motivation ausgewirkt haben dürfte, zielgerichtet zu lernen. Laut Daten des Instituts für Arbeitsmarkt- und Berufsforschung (IAB) nutzten während der Krise lediglich fünf Prozent der Beschäftigten Phasen der Kurzarbeit für Weiterbildung (Bellmann et al. 2021).

Weiterhin wurde in der Krise die zum digitalen Lernen notwendige räumliche und technische Ausstattung ein relevanter Faktor der Weiterbildungskosten. Die erforderlichen Rückzugsmöglichkeiten, das Vorhandensein einer ausreichend schnellen Internetverbindung, die technische Ausstattung und IKT-Kenntnisse waren jedoch unter den Beschäftigten je nach sozio-ökonomischem Status ungleich verteilt (Käpplinger und Lichte 2020).

*Insgesamt* lässt sich mit Blick auf spezifische Arbeitsbedingungen im Zuge der Pandemie nur für das Homeoffice ein überwiegend positiver Effekt auf die Weiterbildungsbeteiligung konstatieren. Kurzarbeit und die Beschäftigung in systemrelevanten Berufen sind hingegen ambivalent zu bewerten; wir erwarten daher keine eindeutigen Effekte auf die Beteiligung an berufsbezogener Weiterbildung während der Pandemie. Darüber hinaus zeigen differenzierte Überlegungen für unterschiedliche Personengruppen, Betriebe und Branchen, dass die Folgen der Pandemie uneinheitlich und komplex sind und sich in deren Verlauf dynamisch entwickelt haben. Daraus geht nicht klar hervor, ob die Beschäftigungsgruppen, die sich üblicherweise besonders wenig oder viel weiterbilden, in der Pandemie in Summe benachteiligt oder bevorteilt wurden. In der Gesamtschau ist daher kaum abzuschätzen, ob die Corona-Krise soziale Ungleichheiten in der Beteiligung an berufsbezogener Weiterbildung nicht beeinflusst hat oder ob sie zu deren Verstärkung oder umgekehrt zu deren Verringerung, d. h. zu größeren oder geringeren Einflüssen der individuellen Voraussetzungen der Beschäftigten, ihrer Beschäftigungsbedingungen sowie betrieblicher Rahmenbedingungen auf die Teilnahme, geführt hat. Von expliziten Hypothesen zu Unterschieden in den Effekten dieser Merkmale über die Zeit sehen wir daher ab und untersuchen die Frage, inwieweit sich die soziale Ungleichheit in der Beteiligung an berufsbezogener Weiterbildung, insbesondere in Abhängigkeit von Bildung und Qualifikationsniveau, im Zuge der Corona-Krise reduziert oder verstärkt hat, explorativ.

## Daten und Methoden

### Datenquellen und Analysestichprobe

Mit Hilfe von multivariaten Regressionsmodellen prüfen wir auf Basis von Längsschnittdaten der Erwachsenenkohorte des Nationalen Bildungspanels (NEPS-Netzwerk 2021; Blossfeld et al. 2011), inwieweit sich die soziale Ungleichheit in der Beteiligung an berufsbezogener Weiterbildung im Zuge der Covid-19 Pandemie verändert hat.^6^ Das NEPS ist die einzige groß angelegte Studie in Deutschland, die detaillierte Längsschnittinformationen zum nonformalen und informellen berufsbezogenen Lernen von erwerbstätigen Erwachsenen erhebt (Janik et al. 2016). Die *Startkohorte Erwachsene* (NEPS-SC6) umfasst Erwachsene der Geburtsjahrgänge 1944–1986, die zum Zeitpunkt der Ziehung in Deutschland lebten. Die Stichprobe wurde zu drei unterschiedlichen Zeitpunkten auf der Basis von Adressen zufällig ausgewählter Einwohnermeldeämter gezogen. Die erste Befragtengruppe wurde im Jahr 2007/08 zum ersten Mal befragt, die zweite im Jahr 2009/10 und die dritte im Jahr 2011/12. Die jährlichen Interviews erfolgen computergestützt, entweder telefonisch oder – insbesondere in den Wellen, in denen Kompetenztests stattfanden – persönlich in den Haushalten der Befragten (für nähere Informationen zu Konzeption und Design vgl. Allmendinger et al. 2019; FDZ-LIfBi 2021).

Um die pandemiebedingten Veränderungen in der BWB zu untersuchen, vergleichen wir die Weiterbildungsaktivitäten derselben Personen vor und während der Corona-Pandemie. Für die Betrachtung von pandemiebedingten Veränderungen verwenden wir Konsortialdaten der Befragungswelle 13, welche von Herbst 2020 bis Frühjahr 2021 erhoben worden sind. Alle Fragen zu Lernaktivitäten im NEPS beziehen sich auf den – i. d. R. knapp einjährigen – Zeitraum seit dem letzten Interview. Folglich bieten die Daten eine ausgezeichnete Grundlage, um erstmals belastbare Aussagen über pandemiebedingte Veränderungen im Zeitraum von Ende 2019 bis Anfang 2021 – also während der ersten sowie der zweiten Welle der Pandemie in Deutschland – auf Basis von Längsschnittdaten zu formulieren. Für den prä-pandemischen Referenzzeitraum unseres Vergleichs nutzen wir vorrangig die Welle 12, die vor Beginn der pandemiebedingten Einschränkungen ab Ende März 2020 abgeschlossen wurde. Diese bildet folglich ausschließlich Weiterbildungsaktivitäten vor der Pandemie, d. h. im Zeitraum Herbst 2019 bis Frühjahr 2020, ab. Für die Beschreibung der veränderten Beteiligung an BWB beziehen wir zusätzlich dazu auch die Welle 11 mit ein, die noch ein Jahr früher, von Herbst 2018 bis Frühjahr 2019, erhoben wurde. Wir tun dies, um zu vermeiden, dass zufällige Schwankungen fälschlicherweise als pandemiebedingter Trend interpretiert werden.

In die Analysen für diesen Beitrag gingen nur Personen ein, die an beiden NEPS-Befragungen teilgenommen haben und die zum Interviewzeitpunkt der Welle 12 *und* vor Beginn der Pandemie erwerbstätig waren. Diese Bedingung ist für den Fokus auf die Teilnahme an berufsbezogener Weiterbildung notwendig, um etwa zu analysieren, wie die veränderte Arbeitssituation während der Corona-Krise (ggf. auch Nichterwerbstätigkeit und Arbeitslosigkeit^7^) den Zugang zu Weiterbildungsgelegenheiten beeinflusst hat. Dazu wurden in Welle 12 zunächst Befragte ausgewählt, die zum Interviewzeitpunkt mit mindestens 5 Wochenstunden im Hauptjob erwerbstätig waren. Im zweiten Schritt wurde die Analysestichprobe weiter auf Befragte beschränkt, die in Welle 13 angegeben haben, vor Beginn der Corona-Pandemie erwerbstätig gewesen zu sein. Und schließlich wurden Personen ausgeschlossen, die zum Zeitpunkt des letzten Interviews über der gesetzlichen Renteneintrittsgrenze lagen.

Die so gebildete Analysestichprobe umfasst insgesamt 4231 Befragte, für die jeweils eine Beobachtung in beiden Erhebungswellen vorliegt. Fehlende Angaben in den unabhängigen Variablen waren sehr selten. Sie wurden, soweit dies möglich war, durch eigene Kategorien in den Modellen berücksichtigt. 28 Personen wurden in den multivariaten Modellen aufgrund fehlender Werte ausgeschlossen; diese resultierten meist aus fehlenden Angaben zur Weiterbildungsstruktur im Betrieb. Damit gingen 4203 Personen in die multivariaten Analysen ein.

Mit dieser Stichprobenauswahl ist es möglich, die Teilnahme an berufsbezogener Weiterbildung bei ein und demselben Personenkreis vor und während der Pandemie zu vergleichen. Verzerrungen aufgrund einer unterschiedlichen Teilnahmeselektivität in den einzelnen Beobachtungsjahren des NEPS können somit eliminiert und Veränderungen in den individuellen Teilnahmeraten zuverlässiger bestimmt werden als bei Vergleichen von Querschnittsdaten. Der Zuschnitt des Analyse-Samples bringt jedoch eine Limitation mit sich, die bei der Interpretation der Ergebnisse berücksichtigt werden muss: Es handelt sich hier um eine selektive Gruppe langjährig Teilnehmender am NEPS, in der bildungshöhere Befragte und Personen ohne Migrationshintergrund überproportional vertreten sind. Alle deskriptiven Ergebnisse werden daher im Folgenden gewichtet dargestellt, um die selektiven Teilnahmeprozesse auszugleichen. In multivariaten Analysen kontrollieren wir relevante Determinanten der Teilnahme dagegen explizit, um keine durch Gewichtung verzerrten Standardfehler zu erzeugen. Dennoch kann nicht ausgeschlossen werden, dass die Beteiligungsraten und die Effekte der untersuchten Determinanten in der Gesamtpopulation anders ausfallen als in unseren Analysestichproben, da eine Selektion in die Befragung auch anhand von unbeobachteten Unterschieden, wie z. B. des intrinsischen Interesses an den Befragungsthemen des NEPS, erfolgt sein mag.^8^

### Analyseverfahren und Operationalisierung

Um die Frage zu beantworten, wie sich die soziale Ungleichheit in der Teilnahme an berufsbezogener Weiterbildung^9^ im Zuge der Covid-19-Pandemie verändert hat, unterscheiden wir die Teilnahme an nonformaler Bildung, also das Lernen in Kursen, von informellen Lernaktivitäten. Für die Analyse der Teilnahme an nonformaler Bildung schätzen wir lineare Wahrscheinlichkeitsmodelle *(linear probability models)* in Form von OLS-Regressionen. Aufgrund der deutlich schieferen Verteilung der abhängigen Variablen für die Beteiligung an informellen Lernaktivitäten schätzen wir logistische Regressionen und berichten durchschnittliche marginale Effekte.

Im NEPS wird die Teilnahme an *nonformaler Bildung* jährlich für den Zeitraum seit dem letzten Interview erfasst. Dazu werden zunächst alle Kurse und Lehrgänge erfasst, die im Kontext aller in diesem Zeitraum stattgefundenen beruflichen Bildungsphasen, Erwerbstätigkeiten, Arbeitslosigkeiten, Elternzeiten und Nichterwerbstätigkeitsphasen stattgefunden haben (zu Details vgl. Janik et al. 2016). Danach wird noch einmal global nach Kursen gefragt, die bis dahin nicht berichtet wurden. Für jeden Kurs wird die Dauer erfasst und es wird erfragt, ob er aus beruflichen oder privaten Gründen aufgenommen wurde. Aus diesen Informationen wurde für die multivariaten Analysen eine Variable zur Abbildung der Beteiligung am *berufsbezogenen nonformalen Lernen* im jeweiligen Jahr gebildet. Diese nimmt den Wert 1 an, wenn mindestens ein Kurs aus beruflichen Gründen besucht wurde.

Daneben werden im NEPS jährlich fünf verschiedene *informelle* Lernaktivitäten erfragt, ebenfalls bezogen auf die Zeit seit dem letzten Interview: (1) der Besuch von Fachmessen oder Kongressen, (2) der Besuch von Fachvorträgen, (3) das Lesen von Fach- und Sachbüchern oder Fachzeitschriften, (4) die Nutzung von Lernprogrammen, Lern-CDs, -DVDs oder Ähnlichem und (5) die Nutzung von Lernangeboten im Internet oder über Apps. Für die Typen (1), (2), (4) und (5) wurde zudem erfragt, ob die letzte Aktivität des jeweiligen Typs aus beruflichen oder privaten Gründen stattgefunden hat. Für die Analysen wurden aus diesen Informationen drei abhängige Indikatoren zur Beteiligung am informellen beruflichen Lernen gebildet, die drei informelle Lernaktivitäten unterscheiden: (1) *Besuch traditioneller Präsenzveranstaltungen*, wie etwa Fachmessen, Kongressen oder Fachvorträgen (Typ 1 und 2), (2) *Lesen von Fachliteratur* (Typ 3) sowie (3) *Nutzung digitaler Medienangebote* (Typ 4 und 5). Auch diese Indikatoren nehmen den Wert 1 an, wenn entsprechend (mindestens) eine informelle Lernform mit beruflicher Nutzung genannt wurde.

Um die spezifischen Erklärungsbeiträge der angebots- und nachfrageseitigen Faktoren für die Beteiligung an berufsbezogener Weiterbildung vor und während der Corona-Pandemie zu vergleichen, nehmen wir die verschiedenen Prädiktoren schrittweise in die Regressionsmodelle auf. Die Verteilung aller Variablen ist in den Tab. A1 (ungewichtet) und A2 (gewichtet) im Online-Appendix für beide Analysejahre dargestellt. Wir berücksichtigen im ersten Schritt zunächst demografische Faktoren wie den Migrationshintergrund (erste und zweite Generation), das Geschlecht, die Haushaltszusammensetzung (Partner und Zahl der Kinder unter 14 Jahren im Haushalt) und die Wohnregion der Befragten (alte/neue Bundesländer), um zunächst die *nachfrageseitigen* Voraussetzungen der Befragten abzubilden. Zusätzlich beziehen wir die beiden Faktoren mit ein, die vorrangig individuelle Investitionskalküle bestimmen: das Alter (in Jahren, linear und quadriert) sowie den höchsten Bildungsabschluss; hier unterscheiden wir vier Bildungsgruppen: (1) keine Ausbildung und Hauptschule mit Ausbildung, (2) Realschule mit Ausbildung, (3) Abitur ohne/mit Ausbildung, (4) Studium.

In den drei nächsten Schritten werden angebotsseitige Opportunitäten und Barrieren berücksichtigt.^10^ Dazu ergänzen wir zunächst *betriebliche Investitionskalküle: *Zentrale Indikatoren dafür sind der berufliche Status der Befragten, den wir über den International Socio-Economic Index (ISEI) erfassen (Ganzeboom et al. 1992), die Arbeitszeit zum Interviewzeitpunkt und die Beschäftigungsdauer im aktuellen Job in Jahren. Als Kontrollvariable wird die Information aufgenommen, ob die Befragten abhängig beschäftigt waren oder nicht.^11^

Im dritten Schritt führen wir die zentralen *Veränderungen der Arbeitssituation während der Corona-Krise* ein. Dies sind der Zugang zu Homeoffice vor der Pandemie sowie während des ersten Lockdowns, ein Dummy zur Betroffenheit von Kurzarbeit oder unbezahlter Freistellung (nur während der Pandemie) sowie ein Dummy für Berufe, die als systemrelevant galten. Diese erfassen wir nach der erweiterten Definition der systemrelevanten Berufe „zweiter Stunde“ von Koebe et al. (2020) auf Basis der Klassifikation der Berufe (KldB 2010, 3‑Steller-Ebene) der Bundesagentur für Arbeit.^12^

Um die *strukturellen Opportunitäten der Betriebe* selbst abzubilden, nehmen wir im vierten Schritt die betriebliche Weiterbildungsstruktur in die Modelle auf. Dazu wurde ein Summenindex mit Werten von 0 bis 4 gebildet, der erfasst, ob es im Betrieb der Befragten (1) eine Betriebsvereinbarung über Weiterbildung, (2) eine regelmäßige Weiterbildungsplanung für die Mitarbeiter/innen sowie (3) eine/n Zuständige/n für Weiterbildung gibt und ob (4) vom Betrieb Kurse oder Lehrgänge angeboten oder finanziert werden.

Für beide Lernaktivitäten interessiert uns vor allem, ob sich die Determinanten der Bildungsbeteiligung im Zuge der Krise verändert haben und ob sich soziale Ungleichheiten vergrößert oder verringert haben. Aus diesem Grund berechnen wir die hierarchischen Regressionsmodelle jeweils getrennt für die letzte Befragungswelle vor Beginn der Pandemie (Herbst/Winter 2019/20) und für die Welle während der Pandemie (Herbst/Winter 2020/21). Die periodenspezifischen Effekte werden grafisch mittels Koeffizienten-Plots der Marginaleffekte dargestellt. Zusätzlich prüfen wir die Differenz der Einflussfaktoren zwischen beiden Wellen in gepoolten Gesamtmodellen mittels Interaktionen mit der Erhebungswelle formal auf statistische Signifikanz.

## Ergebnisse

### Veränderungen der Beteiligung an Weiterbildung

**Figure 1:**
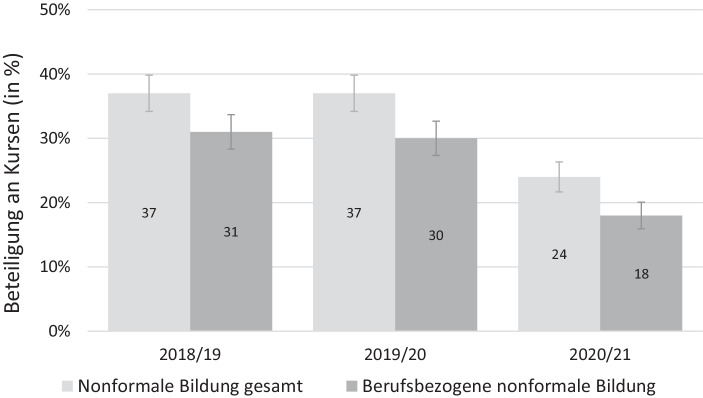

Im ersten Schritt der Ergebnisdarstellung beschreiben wir die Veränderung der Beteiligung an nonformaler und informeller Weiterbildung über die Zeit. Abb. 1 und 2 zeigen die unterschiedlichen Indikatoren für die Beteiligung an nonformaler und informeller BWB bei Teilnehmenden der NEPS-Startkohorte 6, die vor der Pandemie erwerbstätig waren und an den letzten beiden Befragungswellen teilgenommen haben, über einen Zeitraum von drei Jahren. Der letzte Befragungszeitpunkt kennzeichnet die Welle, die vollständig von der Pandemie geprägt war, die beiden früheren lagen vor Beginn der Krise.

**Figure 2:**
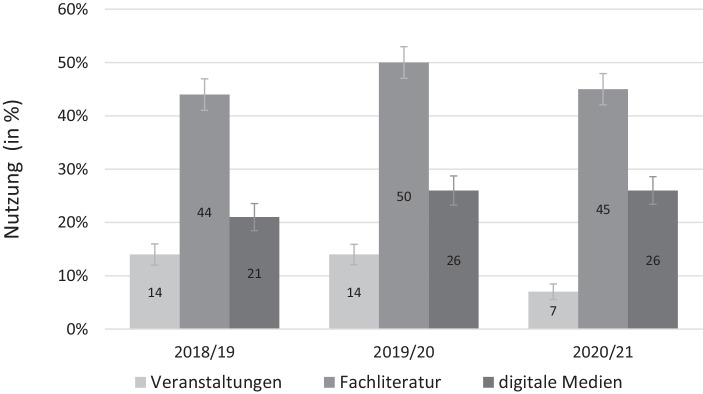

Abb. 1 zeigt, dass die Beteiligung an *nonformaler* berufsbezogener Weiterbildung im Zuge der Pandemie deutlich zurückgegangen ist, während sie zuvor kaum schwankte. Während in den zwei Jahren vor der Pandemie 31 bzw. 30 % der Teilnehmenden Kurse zu beruflichen Zwecken besucht haben, waren es während der Pandemie nur noch 18 %. Diese Verringerung ist statistisch signifikant.

Demgegenüber zeigen die drei Indikatoren für die Beteiligung an *informeller* BWB in Abb. 2 unterschiedliche Veränderungen im Zuge der Pandemie. Insgesamt ist die Beteiligung an Präsenzformen des informellen Lernens, wie der Besuch von Vorträgen und Tagungen, während der Pandemie auf 7 % zurückgegangen, während er in beiden Jahren vor der Pandemie bei konstant 14 % lag. Diese Veränderung ist statistisch signifikant. Das Lesen von Fachliteratur ist in allen drei betrachteten Jahren weit verbreitet. Es stieg vor der Pandemie von 44 % im Jahr 2018/19 auf 50 % im Jahr 2019/20 und fiel während der Pandemie wieder leicht auf 45 %. Dagegen ist die Nutzung digitaler Medien, das heißt das Lernen am Computer und im Internet, von 21 % im Jahr 2018/19 auf 26 % im Jahr 2019/20 gestiegen und während der Pandemie mit 26 % stabil geblieben.

Tab. 1 zeigt die Veränderung in der Beteiligung an nonformaler und informeller BWB während der Corona-Pandemie auf Basis von gepoolten multivariaten Regressionsmodellen ohne und mit vollständigen Kontrollvariablen. Die Befunde bestätigen einen statistisch signifikanten Rückgang der Beteiligung an nonformaler Bildung und der Beteiligung an Präsenzformen sowie des Lesens von Fachliteratur auch unter Kontrolle von zahlreichen Indikatoren auf der individuellen und betrieblichen Ebene.

**Table 1:** 

	(1)		(2)	
	Basis-Modell		vollständiges Modell	
Non-formale berufliche Weiterbildung 2020/21 *(ref. 2019/20)*	−0,12^***^	(0,01)	−0,12^***^	(0,01)
Informelle berufliche Weiterbildung: Konferenzen/Vorträge 2020/21 *(ref. 2019/20)*	−0,12^***^	(0,01)	−0,12^***^	(0,01)
Informelle berufliche Weiterbildung: Lesen 2020/21 *(ref. 2019/20)*	−0,06^***^	(0,01)	−0,07^***^	(0,01)
Informelle berufliche Weiterbildung: (digitale) Medien 2020/21 *(ref. 2019/20)*	−0,00	(0,01)	−0,01	(0,01)
*Kontrollvariablen*	–		✓	

LPM-Modelle für non-formale Weiterbildung sowie average marginal effects auf Basis von Logit-Modellen für informelle Weiterbildung ohne und mit Kontrollvariablen (siehe Tab. A3 im Online Appendix). Standardfehler in Klammern. *N* = 4203

Quelle: NEPS:SC6:12.1.0 (10.5157/NEPS:SC6:12.1.0) & Konsortialdaten B146, eigene Berechnung

^*^p < 0,05, ^**^p < 0,01, ^***^p < 0,001

Insgesamt kann also ein pandemiebedingter Rückgang der Beteiligung am berufsbezogenen Lernen konstatiert werden. Dieser Rückgang war im Bereich nonformaler Weiterbildung und der untersuchten Präsenzform des informellen Lernens stärker ausgeprägt. Zu diesem Trend passen auch Angaben aus der letzten Erhebungswelle 2020/21 – dort berichteten 18 % der Befragten, dass mindestens ein geplanter Weiterbildungskurs aufgrund der Pandemie ausgefallen war. Während sich die Beteiligung an Präsenzveranstaltungen und das Lesen von Fachliteratur etwas verringert hat, hat sich die Beteiligung am digitalen Selbstlernen nicht verändert.

### Die Einflüsse von Voraussetzungen und Opportunitäten vor und während der Pandemie

#### Beteiligung an nonformaler berufsbezogener Weiterbildung

**Figure 3:**
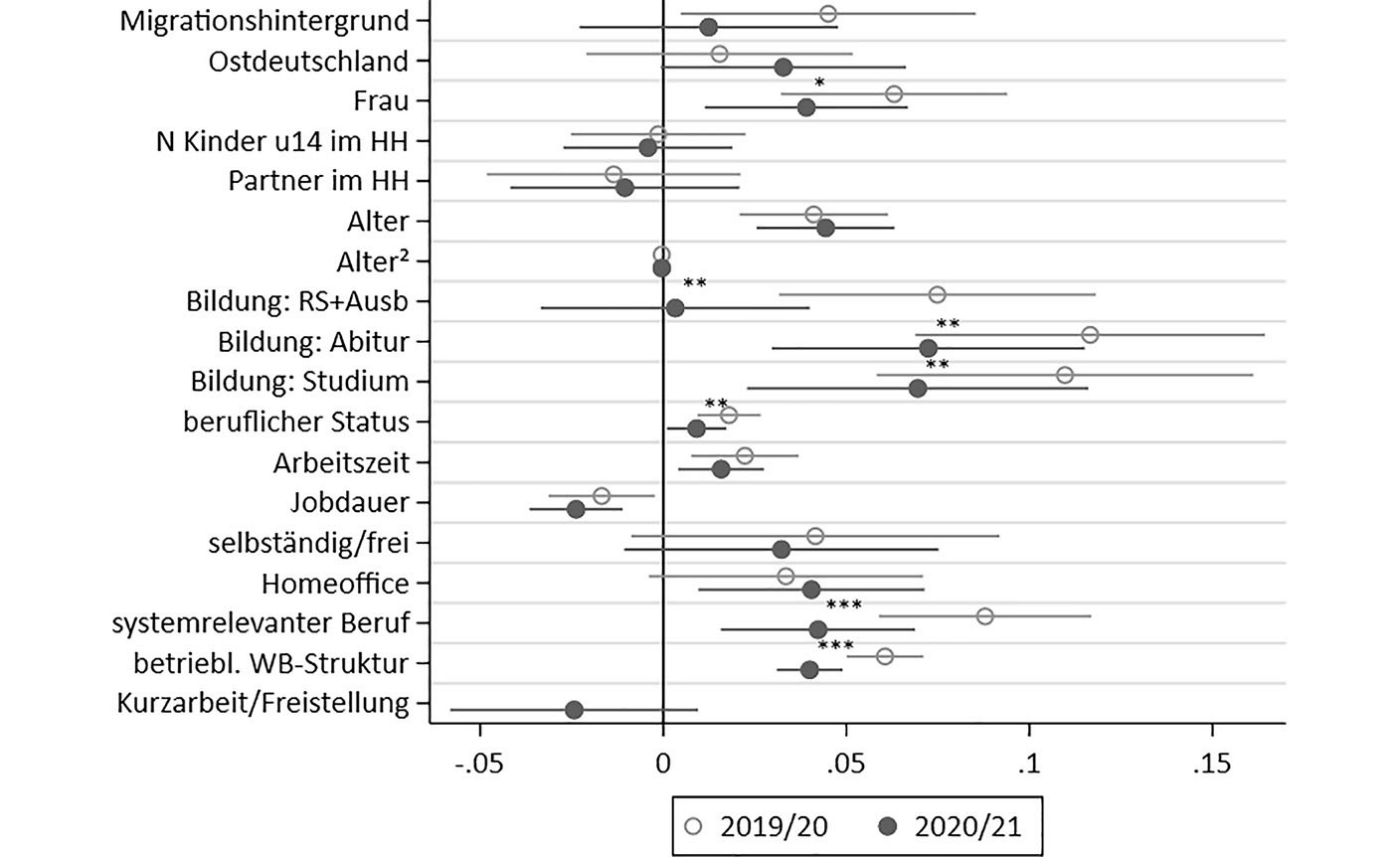

Tab. 2 stellt die Ergebnisse der hierarchisch und nach Erhebungsjahr getrennt geschätzten multivariaten Regression für die Beteiligung am nonformalen berufsbezogenen Lernen dar. Die Modelle 2–4 erweitern das Basismodell mit individuellen Voraussetzungen um die Determinanten der betrieblichen Investitionskalküle (M2), der Arbeitssituation (M3) und der betrieblichen Opportunitätsstrukturen (M4) (siehe Tab. A4 im Online Appendix für vollständige Modelle und Gütekriterien). In Abb. 3 sind die Koeffizienten der vollständigen Modelle mit allen Kontrollvariablen (M4) und deren Konfidenzintervalle für beide Erhebungsjahre zusätzlich grafisch dargestellt. Determinanten, die im gepoolten Modell mit Interaktionseffekt einen statistisch signifikanten periodenspezifischen Einfluss zeigen, sind mittels Sternen gekennzeichnet.

**Table 2:** 

	(1) Individuelle Ressourcen		(2) Betriebliche Investitionskalküle		(3) Arbeitssituation		(4) Betriebliche Ressourcen	
	(1a)	(1b)	(2a)	(2b)	(3a)	(3b)	(4a)	(4b)
	2019/20	2020/21	2019/20	2020/21	2019/20	2020/21	2019/20	2020/21
Migrationshintergrund *(d)*	0,03	0,01	0,04	0,01	0,04	0,01	0,05^*^	0,01
	(0,02)	(0,02)	(0,02)	(0,02)	(0,02)	(0,02)	(0,02)	(0,02)
Ostdeutschland *(d)*	0,01	0,03	0,01	0,03	0,01	0,03	0,02	0,03
	(0,02)	(0,02)	(0,02)	(0,02)	(0,02)	(0,02)	(0,02)	(0,02)
Frau *(d)*	0,05^***^	0,04^**^	0,08^***^	0,05^**^	0,06^***^	0,04^**^	0,06^***^	0,04^**^
	(0,01)	(0,01)	(0,02)	(0,01)	(0,02)	(0,01)	(0,02)	(0,01)
Kinder unter 14 im Haushalt	0,00	0,00	0,00	0,00	0,00	−0,00	−0,00	−0,00
	(0,01)	(0,01)	(0,01)	(0,01)	(0,01)	(0,01)	(0,01)	(0,01)
Partner im Haushalt *(d)*	−0,01	−0,01	−0,01	−0,01	−0,00	−0,01	−0,01	−0,01
	(0,02)	(0,02)	(0,02)	(0,02)	(0,02)	(0,02)	(0,02)	(0,02)
Alter	0,05^***^	0,05^***^	0,05^***^	0,05^***^	0,04^***^	0,05^***^	0,04^***^	0,04^***^
	(0,01)	(0,01)	(0,01)	(0,01)	(0,01)	(0,01)	(0,01)	(0,01)
Alter, quadriert	−0,00^***^	−0,00^***^	−0,00^***^	−0,00^***^	−0,00^***^	−0,00^***^	−0,00^***^	−0,00^***^
	(0,00)	(0,00)	(0,00)	(0,00)	(0,00)	(0,00)	(0,00)	(0,00)
Realschule + Ausbildung	0,13^***^	0,04^*^	0,09^***^	0,02	0,09^***^	0,01	0,08^***^	0,00
	(0,02)	(0,02)	(0,02)	(0,02)	(0,02)	(0,02)	(0,02)	(0,02)
Abitur	0,19^***^	0,13^***^	0,14^***^	0,10^***^	0,14^***^	0,09^***^	0,12^***^	0,07^***^
	(0,02)	(0,02)	(0,02)	(0,02)	(0,02)	(0,02)	(0,02)	(0,02)
Studium	0,23^***^	0,16^***^	0,13^***^	0,09^***^	0,12^***^	0,07^**^	0,11^***^	0,07^**^
	(0,02)	(0,02)	(0,03)	(0,02)	(0,03)	(0,02)	(0,03)	(0,02)
Beruflicher Status (ISEI/10)	–	–	0,03^***^	0,02^***^	0,03^***^	0,01^***^	0,02^***^	0,01^*^
	–	–	(0,00)	(0,00)	(0,00)	(0,00)	(0,00)	(0,00)
Arbeitszeit, in 10 Wo.std.	–	–	0,03^***^	0,02^***^	0,03^***^	0,02^***^	0,02^**^	0,02^**^
	–	–	(0,01)	(0,01)	(0,01)	(0,01)	(0,01)	(0,01)
Jobdauer, in Jahren	–	–	−0,01	−0,02^**^	−0,01	−0,02^**^	−0,02^*^	−0,02^***^
	–	–	(0,01)	(0,01)	(0,01)	(0,01)	(0,01)	(0,01)
Selbständig/freie MA *(d)*	–	–	−0,12^***^	−0,06^***^	−0,12^***^	−0,07^***^	0,04	0,03
	–	–	(0,02)	(0,02)	(0,02)	(0,02)	(0,03)	(0,02)
Zugang zu Homeoffice *(d)*	–	–	–	–	0,04^*^	0,05^***^	0,03	0,04^*^
	–	–	–	–	(0,02)	(0,02)	(0,02)	(0,02)
Systemrelevanter Beruf *(d)*	–	–	–	–	0,11^***^	0,05^***^	0,09^***^	0,04^**^
	–	–	–	–	(0,01)	(0,01)	(0,01)	(0,01)
Kurzarbeit/Freistellung *(d)*	–	–	–	–	–	−0,04^*^	–	−0,02
	–	–	–	–	–	(0,02)	–	(0,02)
Weiterbildungsstruktur	–	–	–	–	–	–	0,06^***^	0,04^***^
Im Betrieb *(d)*	–	–	–	–	–	–	(0,01)	(0,00)
R^2^ (angepasst)	0,03	0,03	0,05	0,04	0,06	0,05	0,09	0,06

Standardfehler in Klammern, *d* für Dummy-Variablen. *N* = 4203

Quelle: NEPS:SC6:12.1.0 (10.5157/NEPS:SC6:12.1.0) & Konsortialdaten B146, eigene Berechnung. Vollständige Modelle in Tab. A4 im Online Appendix

^*^p < 0,05, ^**^p < 0,01, ^***^p < 0,001

Für die individuellen Voraussetzungen zeigen die Ergebnisse in Tab. 2 zunächst, dass Frauen häufiger an nonformaler Bildung teilnahmen als Männer (M1–M4). Diese Vorteile fallen während der Pandemie geringer aus als zuvor (M4), und die Effektstärken vor und während der Pandemie unterscheiden sich unter Kontrolle weiterer betrieblicher und individueller Beschäftigungsmerkmale statistisch signifikant (Abb. 3). Weitere individuelle Merkmale sowie Merkmale der Haushaltszusammensetzung zeigen in allen Modellen einen nur geringen Zusammenhang mit der Teilnahmewahrscheinlichkeit und keine signifikanten Veränderungen über die Zeit.

Individuelle Investitionskalküle sind, wie oben ausgeführt, stark vom Alter sowie vom Bildungsniveau geprägt. Für beide Zeiträume stieg, wie theoretisch zu erwarten, die Weiterbildungsbeteiligung zunächst mit steigendem Alter und sank mit noch höherem wieder ab. Sowohl vor als auch während der Pandemie gilt: Je höher das Bildungsniveau, desto eher beteiligen sich Personen an berufsbezogenen Kursen. Diese Bildungsunterschiede werden etwas geringer, wenn weitere Faktoren in den Modellen kontrolliert werden, sie bleiben aber durchweg signifikant (Tab. 2). Während der Pandemie fielen diese Bildungseffekte geringer aus als zuvor, und die Effektstärken unterscheiden sich signifikant zwischen den beiden Erhebungsjahren (Abb. 3). Insbesondere für Befragte mit Realschulabschluss und Ausbildung war während der Pandemie kein Vorteil in den Weiterbildungschancen mehr gegenüber der Referenzgruppe, Befragte ohne Ausbildung oder mit einem Hauptschulabschluss und Ausbildung, zu erkennen. Das gepoolte Modell bestätigt die statistisch signifikant unterschiedlichen Effektstärken für alle Bildungsgruppen vor und während der Pandemie (Abb. 3). Plottet man die vorhergesagte Beteiligung an nonformaler BWB für die vier Bildungsgruppen, zeigt sich die reduzierte Beteiligung für alle Gruppen, diese ist jedoch für Befragte mit höherem Bildungsniveau besonders stark ausgeprägt (Abb. A1 im Online-Appendix).

Im Bereich betrieblicher Investitionskalküle zeigen vor der Pandemie der berufliche Status und die Arbeitszeit einen positiven Zusammenhang mit der Weiterbildungsbeteiligung. Beide Effekte fielen während der Pandemie etwas schwächer aus, wobei sich nur der Einfluss des beruflichen Status statistisch signifikant über die Zeit unterschied (Abb. 3). Im Gegensatz zu diesen Differenzen verringerte eine längere Dauer der aktuellen Beschäftigung und eine selbstständige Tätigkeit die Teilnahmewahrscheinlichkeit sowohl vor als auch während der Pandemie (Tab. 2, M2 und M3). Obgleich sich die Beteiligung insbesondere für Selbstständige verringerte, waren unter Kontrolle der betrieblichen Weiterbildungsstruktur sowohl die Koeffizienten der periodenspezifischen Regressionen als auch der Interaktionseffekt der Corona-Welle statistisch nicht mehr signifikant (Tab. 2, M4 und Abb. 3). Dies deutet darauf hin, dass in den meist kleinen Betrieben der Selbständigen im Mittel wenig formalisierte Weiterbildungsstrukturen vorhanden sind, was ihre Teilnahme an BVB erschwert.

Während der Pandemie waren drei Aspekte der Arbeitssituation besonders bedeutsam für den Alltag von Erwerbstätigen: die Möglichkeit, von zuhause aus zu arbeiten, die Beschäftigung in einem systemrelevanten Beruf sowie die Betroffenheit von Kurzarbeit. Abb. 3 zeigt, dass der Homeoffice-Zugang und eine Beschäftigung in einem systemrelevanten Beruf positiv mit nonformaler Bildung zusammenhängen – sowohl vor als auch während der Pandemie. Während der positive Zusammenhang beim Homeoffice-Zugang während der Pandemie noch etwas stärker war als zuvor, fielen die Vorteile für Erwerbstätige in systemrelevanten Berufen geringer aus. Der statistisch signifikante Interaktionseffekt im gepoolten Modell bestätigt den geringeren Vorteil während der Pandemie. Kurzarbeit während der Pandemie hatte schließlich einen negativen Effekt auf die Weiterbildungsbeteiligung. Dieser lässt sich jedoch nahezu vollständig durch die Verteilung der Betroffenen über Betriebe mit einer unterschiedlichen Weiterbildungsstruktur erklären (Tab. 2, M4).

Bedeutsame Determinanten für individuelle Bildungschancen sind schließlich auch die betrieblichen Weiterbildungsstrukturen, das heißt, ob Betriebe die Weiterbildung ihrer Beschäftigten planen, Ansprechpartner/innen und eine Betriebsvereinbarung dafür haben und vor allem, ob sie sich an der Finanzierung beteiligen. Auch dieses Merkmal erwies sich während der Krise als weniger bedeutsam für die Erklärung der Bildungsbeteiligung als zuvor (Abb. 3).

Insgesamt erklärten die untersuchten Merkmale die Beteiligungschancen an berufsbezogener nonformaler Bildung während der Pandemie weniger stark als vor der Pandemie, was sich an der geringeren erklärten Varianz der Modelle für das Jahr 2020/21 ablesen lässt (Tab. 2).

#### Beteiligung an informeller berufsbezogener Weiterbildung

**Figure 4:**
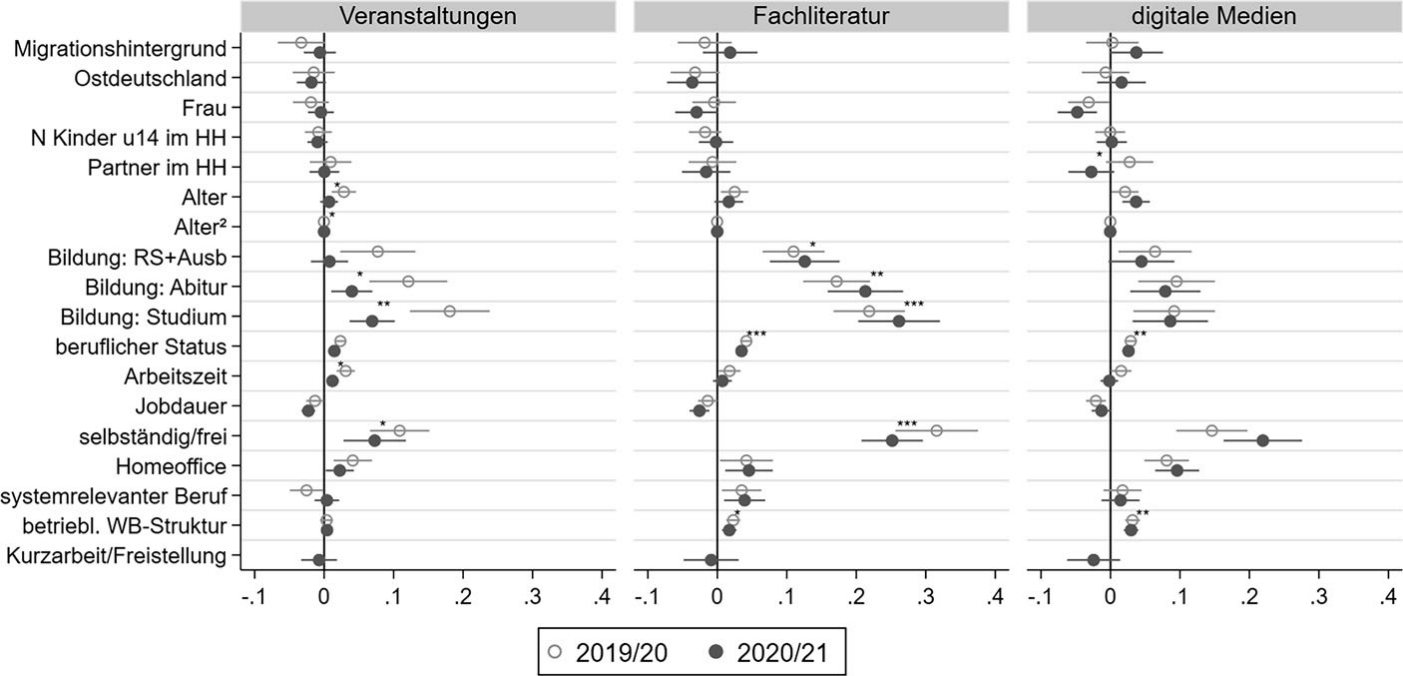

In Abb. 4 sind die marginalen Effekte der logistischen Regressionsmodelle mit einem identischen Aufbau und den gleichen Determinanten zur Erklärung der Beteiligung für alle drei Formen des *informellen* berufsbezogenen Lernens abgebildet (zu den vollständigen Modellen vgl. Tab. A5–A7 im Online-Appendix). Insgesamt zeigen die Ergebnisse, dass sich die Zusammenhänge der untersuchten Merkmale mit dem informellen Lernen ähnlich gestalten wie mit der nonformalen beruflichen Bildung.

Im Gegensatz zur nonformalen Bildung zeigen sich zwischen soziodemographischen Merkmalen und dem informellen Lernen sowohl vor als auch während der Pandemie kaum statistisch signifikante Zusammenhänge. Nur für das Lernen mit digitalen Medien zeigt sich ein statistisch signifikant negativer Zusammenhang für Frauen, welcher sich während der Pandemie noch einmal verstärkt hat. Insgesamt sind die Unterschiede in den periodenspezifischen Einflüssen aller soziodemographischen Merkmale jedoch statistisch nicht signifikant. Allein für den Einfluss des Partners auf das digitale Lernen zeigen sich marginale wellenspezifische Unterschiede, wobei sich der (nicht-signifikante) positive Einfluss vor der Pandemie in einen (ebenfalls nicht signifikanten) negativen Zusammenhang während der Pandemie umkehrt. Zu vermuten ist, dass geringere zeitliche und räumliche Ressourcen in Paarhaushalten für diesen Rückgang verantwortlich sind, welche über die Kontrolle der Zahl von Kindern unter 14 Jahren nur unzureichend erfasst werden.

Im Gegensatz zu soziodemographischen Merkmalen beeinflussen die Indikatoren für individuelle Investitionskalküle, Alter und Bildung, die Teilnahme am informellen Lernen in der theoretisch erwarteten Richtung. Während der Pandemie stand der Besuch von Präsenzveranstaltungen in einem signifikant geringeren Zusammenhang mit dem Lebensalter als zuvor. Zusätzliche Analysen auf Basis vorhergesagter Wahrscheinlichkeiten zeigen, dass die Rückgänge im Besuch von Präsenzveranstaltungen bei den 45–50-Jährigen stärker ausfielen als bei den jüngeren oder älteren Erwerbstätigen (Abb. A5 im Online-Appendix).

Die Zusammenhänge mit dem Bildungsniveau fallen insgesamt ähnlich aus wie beim nonformalen Lernen: Je höher das Bildungsniveau, desto eher lernen Personen informell. Diese Bildungsunterschiede sind für das Lernen über Präsenzveranstaltungen und das Lesen von Fachliteratur deutlich stärker ausgeprägt als für das digitale Lernen. Die pandemiebedingten Veränderungen fielen je nach Form des informellen Lernens unterschiedlich aus: Während die Beteiligung an Präsenzveranstaltungen für alle Bildungsgruppen, insbesondere jedoch für Befragte mit höherem Bildungsniveau, zurückging, verstärkten sich die Bildungsunterschiede beim Lesen von Fachliteratur während der Pandemie. Demgegenüber sind die zeitlichen Unterschiede in der Nutzung digitaler Medien gering und statistisch nicht signifikant. Die vorhergesagte Beteiligung am informellen Lernen nach Bildungsniveau bestätigt die statistisch signifikant verringerte Nutzung von Präsenzveranstaltungen, welche für Personen mit höherem Bildungsniveau besonders ausgeprägt war. Für das Lesen von Fachliteratur gehen die gestiegenen Bildungsungleichheiten vor allem auf eine signifikant verringerte Nutzung bei Befragten mit Realschulabschluss und Ausbildung zurück (Abb. A2 im Online-Appendix).

Im Bereich betrieblicher Investitionskalküle zeigen sich für die meisten Determinanten statistisch signifikante Zusammenhänge mit dem informellen Lernen vor der Pandemie (Abb. 4). Insbesondere der berufliche Status, die Arbeitszeit und das Ausüben einer selbstständigen Tätigkeit beeinflussten das informelle Lernen positiv, während eine zunehmende Jobdauer mit einer geringeren Nutzungswahrscheinlichkeit korrelierte. Gepoolte Modelle zeigten einen statistisch signifikant verringerten Einfluss des beruflichen Status auf das Lernen mittels Fachliteratur und digitalen Medien sowie der Arbeitszeit auf das Lernen in Präsenzveranstaltungen. Während sich der positive Zusammenhang zwischen einer selbstständigen Tätigkeit und der Teilnahme an Präsenzveranstaltungen sowie dem Lesen von Fachliteratur während der Pandemie verringerte, verstärkte sich der Zusammenhang für die Nutzung digitaler Medien leicht. Allerdings bestätigten gepoolte Modelle die statistisch signifikante Veränderung vor dem Hintergrund der kleinen Teilgruppe von Selbstständigen nur für die Teilnahme an Präsenzveranstaltungen und das Lesen von Fachliteratur.

Auch im Bereich der veränderten Arbeitssituation während der Corona-Krise unterscheiden sich die Befunde für das informelle Lernen von jenen für das nonformale Lernen. Zwar hatten Beschäftigte mit Zugang zu Homeoffice erneut deutliche Vorteile gegenüber Beschäftigten, die nicht von zuhause aus arbeiten konnten. Diese Unterschiede fielen jedoch – im Gegensatz zu nonformaler Weiterbildung – während der Pandemie ähnlich aus wie zuvor. Ebenfalls nur geringfügig veränderte sich die Beteiligung von Beschäftigten in systemrelevanten Berufen, die nur mit Blick auf das Lesen von Fachliteratur einen Vorteil gegenüber anderen Berufsgruppen hatten. Auch dieser Befund steht im Gegensatz zum Einfluss dieses Merkmals auf das nonformale Lernen. Die Betroffenheit von Kurzarbeit während der Pandemie hatte, ebenso wie beim nonformalen Lernen, einen tendenziell negativen Effekt auch auf das informelle Lernen, wobei der Effekt in keinem Modell statistisch signifikant ist.

Auch für das informelle Lernen scheinen betriebliche Weiterbildungsstrukturen relevant zu sein. Entsprechend positiv ist der Zusammenhang mit den drei Indikatoren des informellen beruflichen Lernens. Ähnlich wie bei der nonformalen Weiterbildung erwies sich der Zusammenhang während der Krise als signifikant weniger bedeutsam für die Erklärung des Lesens von Fachliteratur und der Nutzung digitaler Medien (Abb. 4).

### Sensitivitätsanalysen

In zusätzlichen Sensitivitätsanalysen testen wir den Einfluss der Branchenstruktur der Betriebe, in denen die Befragten beschäftigt waren. Da die Branchenstruktur eng mit Merkmalen der Arbeitssituation sowie der Opportunitätsstrukturen der Betriebe – insbesondere im Zeitraum der Pandemie – zusammenhängt, ist das Problem einer Überkontrolle, die eine Verzerrung der Effekte dieser Merkmale bewirken würde, wahrscheinlich. Aus diesem Grund testen wir den Einfluss der Branchenstruktur in den vollständigen Modellen nur ergänzend. Dazu unterscheiden wir 20 Wirtschaftszweige basierend auf der Ebene der Abschnitte in der Klassifikation der Wirtschaftszweige von 2008. Die Ergebnisse dieses Modells zeigen, dass die statistisch signifikanten Rückgänge im nonformalen und informellen berufsbezogenen Lernen (Besuch von Veranstaltungen und Lesen von Fachliteratur) auch unter Kontrolle der Branchenstruktur bestehen bleiben (siehe Tab. A3 im Online-Appendix, M3). Darüber hinaus unterscheiden sich die dargestellten Ergebnisse der multivariaten Periodenvergleiche nur geringfügig und nur für einzelne erwartbare Determinanten, etwa das Geschlecht oder die pandemiebedingt veränderte Arbeitssituation (siehe Tab. A4–A7 im Online-Appendix, M5).

Ergänzend wurden alternative Operationalisierungen der unabhängigen Variablen sowie nicht-lineare Effekte mittels Robustheitsprüfungen geprüft. Um zu berücksichtigen, dass in der Pandemie vor allem Mütter die Betreuung von Kindern übernommen haben (Zoch et al. 2021), haben wir einen Interaktionseffekt für den geschlechtsspezifischen Effekt von Kindern unter 14 Jahren im Haushalt getestet. Die Ergebnisse zeigen statistisch nicht signifikante Interaktionseffekte, das heißt vergleichbare Verringerungen im nonformalen und informellen Lernen bei Männern und Frauen ohne und mit Kindern unter 14 Jahren im Haushalt. Dies dürfte vermutlich auf das vergleichsweise hohe Alter und die entsprechend geringe Anzahl an jungen Kindern in der NEPS-Stichprobe zurückzuführen sein (Abb. A5 und A6 im Online-Appendix). Die geringen Zellbesetzungen erlauben daher auch keine Modellspezifikationen, welche die Altersgruppe des jüngsten Kindes berücksichtigen würden.

Um abschließend auch Veränderungen in der Intensität der nonformalen beruflichen Weiterbildung zu betrachten, untersuchen wir explorativ für diejenigen Befragten, die an berufsbezogenen Kursen teilgenommen haben, wie sich die kumulierte Dauer aller Kurse in Stunden während der Krise entwickelt hat. Die Ergebnisse zeigen, dass sich für die Gruppe von Teilnehmenden die Gesamtdauer aller beruflich motivierten Kurse kaum verändert hat (Abb. A7 im Online-Appendix).

## Zusammenfassung und Diskussion

Die vorliegende Studie zeigt auf Basis der Längsschnittdaten der NEPS-Startkohorte 6, dass die Covid-19-Pandemie mit einem moderaten *Rückgang der Teilnahme *an berufsbezogener Weiterbildung in Deutschland einherging. Dabei fiel der Rückgang im Bereich nonformaler Weiterbildung und für informelles Lernen in Präsenzveranstaltungen etwas stärker aus als für das informelle Lernen mit analogen oder digitalen Medien. Während sich das Lesen von Fachliteratur moderat verringerte, blieb das Lernen am Computer und im Internet während der Pandemie weitestgehend stabil. Die Krise hat demnach trotz der fehlenden Lernmöglichkeiten vor Ort und der Zurückhaltung vieler Betriebe in Weiterbildungsinvestitionen nicht zu einem Zusammenbruch der Beteiligung an Weiterbildung geführt, was auch damit zusammenhängen dürfte, dass es für viele Erwerbstätige notwendig war, in kurzer Zeit neue Dinge zu erlernen, insbesondere in digitaler Form. Vor diesem Hintergrund erscheint der Befund, dass sich das digitale Lernen während der Pandemie nicht intensiviert hat, sondern stabil geblieben ist, zunächst überraschend. Erklärungen dafür könnten erstens darin liegen, dass das digitale Ad-hoc-Lernen zu Beginn der Pandemie mit den beiden Indikatoren im NEPS nicht gut abgebildet wird. Darauf deuten die hohen Anteilswerte von Befragten hin, die angaben, dass Weiterbildungen während der Corona-Krise auf Online-Formate umgestellt wurden. Zweitens ist nicht auszuschließen, dass gerade im ersten Krisenjahr zeitliche Restriktionen und mangelnde Motivation, die durch subjektive Unsicherheit entstand, Hürden für intensiveres digitales Lernen dargestellt haben. Zudem könnten sich Erwerbstätige in diesem Zeitraum auf andere Lebensinhalte und -bereiche wie Familie und Gesundheit fokussiert haben. Überprüfen lassen sich diese Annahmen mit unseren Daten allerdings nicht.

Die multivariaten Ergebnisse der Studie zeigen insgesamt, dass die *Auswirkungen der Pandemie *auf die Beteiligung an berufsbezogener Weiterbildung komplex sind. So zeigt sich zwar eine Tendenz zu einer sozial weniger ungleichen Beteiligung an der nonformalen berufsbezogenen Weiterbildung sowie am informellen Lernen in Präsenzveranstaltungen. Insgesamt sind die stark ausgeprägten sozialen, beruflichen und betrieblichen Differenzen allerdings nur leicht zurückgegangen; das heißt, die Rückgänge in der Beteiligung fielen bei denjenigen, die sich normalerweise sehr stark an Weiterbildung beteiligen, etwas höher aus als bei denjenigen, die das weniger tun. Dies gilt insbesondere für hoch Gebildete, Personen mit hohem beruflichen Status, Vollzeiterwerbstätige und Beschäftigte in weiterbildungsstarken Betrieben. Im Gegensatz dazu haben sich die bereits zuvor ausgeprägten Bildungsungleichheiten beim Lesen von Fachliteratur während der Krise sogar signifikant vergrößert. Und schließlich blieben die geringer ausgeprägten Bildungsunterschiede beim Lernen mit digitalen Medien unverändert.

Für diese Befunde gibt es unterschiedliche Interpretationsmöglichkeiten: Erstens könnten die weiterbildungsintensiven Beschäftigtengruppen während der Krise besonders wenig Zeit für organisierte Weiterbildung sowie für die Teilnahme an Veranstaltungen gehabt haben, zum Beispiel, weil sie mehr Zeit und Ressourcen für das Homeschooling ihrer Kinder aufgewendet haben oder die Arbeitsbelastung im Job während der Krise gestiegen ist. Das Lesen von Fachliteratur ist dagegen zeitlich flexibler, so dass insbesondere diese Beschäftigtengruppen nicht vorhandene oder nicht realisierbare Weiterbildungsmöglichkeiten möglicherweise durch das Lesen von Fachliteratur kompensiert haben. Zweitens liegt die Erklärung nahe, dass sich die Inhalte von Weiterbildung während der Krise verändert haben. Viele Beschäftige mussten während der Pandemie lernen, digitale Kollaborationstools oder Lernplattformen zu bedienen, oder neue Regelungen zum Arbeitsschutz umzusetzen. Solche Lerninhalte betrafen sehr große Gruppen von Erwerbstätigen, darunter auch viele in normalerweise wenig weiterbildungsintensiven Tätigkeiten. Drittens ist nicht auszuschließen, dass sich hinter dem Rückgang der Ungleichheiten nur ein statistisches Artefakt verbirgt, nämlich eine Art Bodeneffekt. Darauf deutet der Befund hin, dass sich die sozialen Unterschiede nur bei denjenigen Weiterbildungsformen verringert haben, die während der Pandemie nicht oder nur mit Auflagen durchführbar waren und deren Umstellung auf digitale Formate aufwendig und teuer ist.

Auch die Geschlechterdifferenzen in der Beteiligung an berufsbezogener Weiterbildung haben sich während der Krise verändert, und zwar durchgehend zuungunsten von Frauen. So ging der Vorteil von Frauen gegenüber Männern bei der Beteiligung an nonformaler Bildung während der Krise signifikant zurück, und ihr Nachteil beim Lernen mit digitalen Medien hat sich während der Pandemie noch einmal verstärkt. Dass sich erwerbstätige Frauen insgesamt etwas stärker an Weiterbildung beteiligen als erwerbstätige Männer, zeigt nicht nur das NEPS, sondern auch der AES (BMBF 2021, S. 39). Im NEPS ist der Vorteil von Frauen zusätzlich dadurch zu erklären, dass die Befragten eher älter sind und nur noch zu geringen Anteilen jüngere Kinder im Haushalt haben. Dass der Rückgang der Beteiligung an Weiterbildung bei Frauen in der Krise etwas stärker ausgefallen als bei Männern, mag auf ihre insgesamt stärkeren zeitlichen Belastungen, z. B. durch Hausarbeit, Homeschooling, aber auch auf ihre starke Repräsentanz in systemrelevanten Berufen zurückzuführen sein.

Im Gegensatz zu den zeitunveränderlichen sozialen, beruflichen und betrieblichen Gruppenunterschieden hatte die *veränderte Arbeitssituation *während der Corona-Krise differenzielle und teilweise neue Einflüsse. Beschäftigte in systemrelevanten Berufen beteiligten sich vor der Krise besonders stark an nonformaler Weiterbildung. Während der Pandemie fiel dieser Vorteil geringer aus als davor. Dies könnte ein Hinweis auf besondere zeitliche Belastungen dieser Beschäftigtengruppe sein, die weniger Zeit für Fortbildungen ließen. Beschäftigte mit Zugang zu Homeoffice beteiligten sich vor der Krise intensiver als andere am informellen, insbesondere am digitalen Lernen. Tendenziell galt das auch für nonformale Bildung. Während der Krise hatten plötzlich viel mehr Beschäftigte als zuvor die Möglichkeit, von zuhause aus zu arbeiten. Diese Gruppe konnte ihren Vorteil bei der Beteiligung am nonformalen Lernen ausbauen, die Vorteile im Bereich des informellen Lernens veränderten sich dagegen nicht. Vor dem Hintergrund der Tatsache, dass digitales Lernen wichtiger geworden ist, könnte der Zugang zu Homeoffice künftig ein weiterer ungleichheitsgenerierender Faktor für das nonformale Lernen im Erwachsenenalter sein, dessen weitere Entwicklung es zu beobachten gilt. Personen, die während der Pandemie von Kurzarbeit betroffen waren, lernten tendenziell weniger als andere nonformal sowie informell, wobei die Differenzen bei keiner Lernform signifikant waren. Dies könnte einerseits damit zu erklären sein, dass die Betriebe, in denen sie beschäftigt waren, über weniger Ressourcen für Weiterbildung verfügten als andere, weil sie durch die Krise besonders stark belastet waren. Andererseits kann Kurzarbeit auch für die betroffenen Beschäftigten selbst subjektiv belastend und somit motivationshemmend für das Lernen sein. Insgesamt deuten unsere Ergebnisse somit darauf hin, dass mit dem Arbeiten von zuhause aus tatsächlich neue Spielräume für berufsbezogene Weiterbildung entstanden sind, nicht jedoch mit der Kurzarbeit.

Es ist die Aufgabe künftiger Forschung zu beobachten, wie sich die beschriebenen Trends weiterentwickeln, die offen gebliebenen Interpretationsangebote zu überprüfen und die Limitationen unserer Studie zu überwinden. Vier Aspekte erscheinen uns hier besonders fruchtbar: Erstens könnten künftige Studien auf Basis von Längsschnittdaten und Methoden der Kausalanalyse stärker für unbeobachtete Heterogenität kontrollieren. Wir können hingegen nicht ausschließen, dass unsere Ergebnisse aufgrund von unbeobachteter Heterogenität sowie Selektivität aufgrund von Panelattrition verzerrt sind, auch wenn wir versucht haben, dies durch die Aufnahme geeigneter Variablen von denen wir wissen, dass sie die Teilnahme am Survey sowie an der BWB beeinflussen, auszugleichen. Zweitens könnten in künftigen Analysen auch die Lerninhalte stärker analysiert werden, um beispielsweise die Frage nach ‚trade-offs‘ zwischen umfangreichen Präsenzfortbildungen und kurzen digitalen Lerneinheiten besser zu beleuchten, als wir das konnten. Drittens erscheint es sinnvoll, die Finanzierungsart vor allem von nonformaler Weiterbildung stärker in den Blick zu nehmen, als wir es mit unseren Daten konnten, um damit Auswirkungen der Pandemie auf unterschiedliche Weiterbildungssegmente (Friebel 1993a, b) zu untersuchen. Viertens könnte künftige Forschung auch davon profitieren, wenn sowohl der zeitliche Verlauf der Pandemie als auch unterschiedliche regionale Problemlagen explizit in den Analysen berücksichtigt werden würden. Damit ließe sich exakter lokalisieren, welche Auswirkungen die Pandemie auf unterschiedliche Beschäftigtengruppen bis heute hat.

## Supplementary Information





## Footnotes

1 Damit beziehen wir uns auf die Tatsache, dass es im Bereich nonformaler und informeller Weiterbildung keine universellen gesellschaftlichen Einrichtungen wie Schulen oder Universitäten gibt, in denen Bildungsprozesse und deren Ergebnisse verpflichtend geregelt werden, woraus sich Erwartungszwänge und Handlungsverpflichtungen ergeben. Gleichwohl gibt es auch hier institutionelle Rückbezüge, zum Beispiel an die betrieblich organisierte Arbeit oder an Fördermaßnahmen im Zuge aktiver Arbeitsmarktpolitik.

2 Wir konzentrieren uns im Folgenden auf ausgewählte informelle Lernaktivitäten, welche im NEPS erfasst werden. Diese beziehen sich entweder auf digitales Lernen (Nutzung von Selbstlernprogrammen und Lernangeboten im Internet) sowie auf Fachkommunikation (Besuch von Fachmessen, Kongressen, Vorträgen, Lesen von Fachliteratur) (zu dieser Systematisierung vgl. Kuper und Kaufmann 2010).

3 Im AES zählen nicht nur Kurse und Lehrgänge zur Weiterbildung, sondern auch kurzzeitige Bildungsveranstaltungen wie Vorträge, Seminare und Workshops, Schulungen am Arbeitsplatz und Privatunterricht in der Freizeit.

4 Dabei wurde nicht zwischen berufsbezogener und privater Nutzung unterschieden.

5 Jenseits dessen gibt es auch eine betriebliche Nachfrage nach Weiterbildungsangeboten, z. B. wenn diese von externen Bildungsanbietern eingekauft werden. Diese Ebene von Angebot und Nachfrage nehmen wir in unserer Studie aber nicht in den Blick.

6 Diese Arbeit nutzt Daten des Nationalen Bildungspanels (NEPS): Startkohorte Erwachsene, 10.5157/NEPS:SC6:12.0.1 sowie bislang unveröffentlichte Daten einer späteren Welle dieser Startkohorte. Die Daten des NEPS wurden von 2008 bis 2013 als Teil des Rahmenprogramms zur Förderung der empirischen Bildungsforschung erhoben, welches vom Bundesministerium für Bildung und Forschung (BMBF) finanziert wurde. Seit 2014 wird das NEPS vom Leibniz-Institut für Bildungsverläufe (LIfBi) in Kooperation mit einem deutschlandweiten Netzwerk weitergeführt.

7 Diese Gruppen sind jedoch sehr klein: Zum Interviewzeitpunkt der Welle 13 gaben 151 Personen in der Analysestichprobe an, aktuell nicht mehr erwerbstätig zu sein (3,6 % der Befragten), darunter waren 49 Personen (1,2 % der Befragten) arbeitslos.

8 Auf die alternative Strategie einer multiplen Imputation der Informationen für fehlende Teilnahmewellen wurde bewusst verzichtet, da diese eine konstante Schätzung der Teilnahme an berufsbezogener Weiterbildung über die Zeit (sowie aller anderen in den Analysen verwendeten Informationen) unterstellt. Genau diese Annahme ist aber pandemiebedingt vermutlich nicht zutreffend.

9 Zu beachten ist dabei, dass in den NEPS-Daten betriebliche Weiterbildung i. S. v. arbeitgeberfinanzierten Maßnahmen weder eindeutig identifiziert noch von mischfinanzierter und arbeitnehmerfinanzierter Weiterbildung unterschieden werden kann, weil diese Informationen nur für einen Teil der erfassten nonformalen Bildung vorliegen.

10 Aufgrund der Tatsache, dass es zum Zeitpunkt der Datenauswertung für die letzte Erhebungswelle noch keinen Scientific Use File gab, sind einige Einschränkungen zu beachten. So waren Informationen zum aktuellen Beruf und Wirtschaftszweig noch nicht codiert. Diese wurden durch die Angaben der Vorwelle ersetzt.

11 Alle Jobmerkmale beziehen sich auf die Haupterwerbstätigkeit zum Interviewzeitpunkt. Für abhängig Beschäftigte wird die vertragliche, für Selbständige und Saisonarbeiter/innen die tatsächliche Arbeitszeit benutzt. Personen, die zum Zeitpunkt des letzten Interviews nicht mehr erwerbstätig waren, werden dadurch kontrolliert, dass bei ihnen die Arbeitszeit auf 0 gesetzt wird. Um diese Gruppe nicht ausschließen zu müssen, wird bei ihnen die Beschäftigungsdauer der Haupterwerbstätigkeit im Jahr zuvor um die Zeit seit dem letzten Interview verlängert. Für alle übrigen Beschäftigungsmerkmale (abhängige vs. selbständige Beschäftigung, betriebliche Weiterbildungsstruktur) werden für diese Gruppe die Angaben aus der Vorwelle benutzt.

12 Die Liste systemrelevanter Berufe wurde für die Festlegung des Anspruchs auf Kindernotbetreuung auf Länderebene festgelegt. Obgleich keine bundesweit einheitliche Liste existiert, unterscheiden sich die Länderlisten hinsichtlich der Berufsgruppen nicht grundsätzlich. Für einen nachvollziehbaren Vergleich mit relevanten Vorarbeiten orientiert sich die vorliegende Analyse an der sog. Berliner Liste vom 27. April 2020 (Koebe et al. 2020).

## References

[CR1] Allmendinger J, Kleinert C, Pollak R, Vicari B, Wölfel O, Althaber A, Antoni M, Christoph B, Drasch K, Janik F, Künster R, Laible M-C, Leuze K, Matthes B, Ruland M, Schulz B, Trahms A, Blossfeld H-P, Roßbach H-G (2019). Adult education and lifelong learning. Adult education as a lifelong process. The German national educational panel study (NEPS).

[CR2] Becker R, Abraham M, Hinz T (2018). Berufliche Weiterbildung im Arbeitsmarkt. Arbeitsmarktsoziologie. Probleme, Theorien, empirische Befunde.

[CR3] Beicht U, Walden G (2006). Individuelle Investitionen in berufliche Weiterbildung – Heutiger Stand und künftige Anforderungen. WSI Mitteilungen.

[CR4] Bellmann, L., Gleiser, P., Kagerl, C., Koch, T., König, C., Kruppe, T., Lang, J., Leber, U., Pohlan, L., Roth, D., Schierholz, M., Stegmaier, J., & Aminian, A. (2020). Weiterbildung in der Covid-19-Pandemie stellt viele Betriebe vor Schwierigkeiten. *IAB-Forum 09.12.2020*. Nürnberg. https://www.iab-forum.de/weiterbildung-in-der-covid-19-pandemie-stellt-viele-betriebe-vor-schwierigkeiten/. Zugegriffen: 22. Nov. 2021.

[CR5] Bellmann, L., Kruppe, T., & Segert-Hess, N. (2021). Qualifizierung während Corona: Wie stark nutzen Betriebe Kurzarbeit für Weiterbildungen? *IAB-Forum 17.05.2021*. Nürnberg. https://www.iab-forum.de/qualifizierung-waehrend-corona-wie-stark-nutzen-betriebe-kurzarbeit-fuer-weiterbildungen/?pdf=22747. Zugegriffen: 22. Nov. 2021.

[CR6] Blossfeld, H.-P., Roßbach, H.-G., & von Maurice, J. (Hrsg.). (2011). *Education as a Lifelong Process. The German National Educational Panel Study* (Zeitschrift für Erziehungswissenschaft: Sonderheft 14). Wiesbaden: Springer VS.

[CR7] BMBF (2021). *Weiterbildungsverhalten in Deutschland 2020. Ergebnisse des Adult Education Survey – AES-Trendbericht*. Berlin. https://www.bmbf.de/SharedDocs/Publikationen/de/bmbf/1/31690_AES-Trendbericht_2020.html. Zugegriffen: 27. Juni 2022.

[CR8] Bundesinstitut für Berufsbildung (2021). Weiterbildungsbranche von Corona schwer getroffen. Erste Ergebnisse der wbmonitor-Umfrage 2020 von BIBB und DIE.

[CR9] Denninger A, Käpplinger B (2021). COVID-19 und Weiterbildung – Überblick zu Forschungsbefunden und Desideraten. Zeitschrift für Weiterbildungsforschung.

[CR10] Dieckhoff M, Steiber N (2011). A re-assessment of common theoretical approaches to explain gender differences in continuing training participation. British Journal of Industrial Relations.

[CR11] Eccles JS, Elliot AJ, Dweck CS (2005). Subjective task values and the Eccles et al. model of achievement related choices. Handbook of competence and motivation.

[CR12] Ehlert, M., Hornberg, C., & Scholl, F. (2020). *Weiterbildung in der Krise? Herausforderungen und Chancen für das lebenslange Lernen durch COVID-19. Corona und die gesellschaftlichen Folgen: Schlaglichter aus der WZB-Forschung*. Berlin. https://www.econstor.eu/handle/10419/223154. Zugegriffen: 22. Nov. 2021.

[CR13] Euler D, Severing E (2020). Nach der Pandemie: für eine gestaltungsorientierte Berufsbildung in der digitalen Arbeitswelt. Eine Denkschrift.

[CR14] FDZ-LIfBi (2021). Data Manual: NEPS Starting Cohort 6—Adults: Adult Education and Lifelong Learning. Scientific Use File Version 12.0.1. Bamberg. https://www.neps-data.de/Portals/0/NEPS/Datenzentrum/Forschungsdaten/SC6/12-0-1/SC6_12-0-1_Datamanual.pdf. Zugegriffen: 20. Juni 2022.

[CR15] Flake, R., Seyda, S., & Werner, D. (2020). Weiterbildung während der Corona-Pandemie. *KOFA kompakt*. https://bildungsklick.de/fileadmin/user_upload/www.bildungsklick.de/PDFs/Weiterbildung_waehrend_Corona-Pandemie.pdf. Zugegriffen: 22. Nov. 2021.

[CR16] Friebel H (1993). Individuelle und institutionelle Akteure der Weiterbildung. Zeitschrift für Berufs- und Wirtschaftspädagogik.

[CR17] Friebel H, Friebel H (1993). Der gespaltene Weiterbildungsmarkt und die Lebenszusammenhänge der Teilnehmer/-innen. Weiterbildungsmarkt und Lebenszusammenhang.

[CR18] Frodermann, C., Grunau, P., Haepp, T., Mackeben, J., Ruf, K., Steffes, S., & Wanger, S. (2020). Online-Befragung von Beschäftigten: Wie Corona den Arbeitsalltag verändert hat. *IAB-Kurzbericht 13/2020*. Nürnberg. https://doku.iab.de/kurzber/2020/kb1320.pdf. Zugegriffen: 22. Nov. 2021.

[CR19] Ganzeboom HBG, de Graaf PM, Treiman DJ (1992). A standard international socio-economic index of occupational status. Social Science Research.

[CR20] Gorges J (2015). Warum (nicht) an Weiterbildung teilnehmen?. Zeitschrift für Erziehungswissenschaft.

[CR21] International Labour Organization (2021). *Skilling, upskilling and reskilling of employees, apprentices & interns during the COVID-19 pandemic. Findings from a global survey of enterprises*. Geneva. https://www.ilo.org/wcmsp5/groups/public/---ed_emp/---emp_ent/documents/publication/wcms_794569.pdf. Zugegriffen: 27. Juni 2022.

[CR22] Janik F, Wölfel O, Trepesch M, Blossfeld H-P, von Maurice J, Bayer M, Skopek J (2016). Measurement of further training activities in life-course studies. Methodological issues of longitudinal surveys. The example of the national educational panel study.

[CR23] Jost, R., & Leber, U. (2021). Die betriebliche Weiterbildung ist in der Corona-Krise massiv eingebrochen. *IAB-Forum 10.12.2021*. Nürnberg. https://www.iab-forum.de/die-betriebliche-weiterbildung-ist-in-der-corona-krise-massiv-eingebrochen/. Zugegriffen: 27. Juni 2022.

[CR24] Käpplinger B, Lichte N (2020). “The lockdown of physical co-operation touches the heart of adult education”: a Delphi study on immediate and expected effects of COVID-19. International Review of Education.

[CR25] Kaufmann K, Rohs M (2016). Beteiligung am informellen Lernen. Handbuch Informelles Lernen.

[CR26] Kaufmann K, Widany S (2013). Berufliche Weiterbildung – Gelegenheits- und Teilnahmestrukturen. Zeitschrift für Erziehungswissenschaft.

[CR27] Kilpi-Jakonen E, Vono de Vilhena D, Blossfeld H-P (2015). Adult learning and social inequalities: processes of equalisation or cumulative disadvantage?. International Review of Education.

[CR29] Kleinert C, Wölfel O (2018). Technologischer Wandel und Weiterbildungsteilnahme. Berufsbildung in Wissenschaft und Praxis.

[CR28] Kleinert, C., Bächmann, A.-C., Schulz, B., Vicari, B., & Ehlert, M. (2021a). Für wen brachte Corona einen Digitalisierungsschub? Veränderungen in der Nutzung digitaler Technologien während der COVID-19-Pandemie. *NEPS Corona & Bildung 6*. Bamberg. https://www.lifbi.de/Portals/13/Corona/NEPS_Corona-und-Bildung_Bericht_6-Digitalisierung.pdf. Zugegriffen: 27. Juni 2022.

[CR30] Kleinert C, Zoch G, Vicari B, Ehlert M (2021). Work-related online learning during the COVID-19 pandemic in Germany. Zeitschrift für Weiterbildungsforschung.

[CR31] Koebe, J., Samtleben, C., Schrenker, A., & Zucco, A. (2020). Systemrelevant, aber dennoch kaum anerkannt: Entlohnung unverzichtbarer Berufe in der Corona-Krise unterdurchschnittlich. *DIW aktuell 48*. Berlin. https://www.diw.de/documents/publikationen/73/diw_01.c.792728.de/diw_aktuell_48.pdf. Zugegriffen: 22. Nov. 2021.

[CR32] Kuper H, Kaufmann K (2010). Beteiligung an informellem Lernen. Zeitschrift für Erziehungswissenschaft.

[CR33] Kuwan H, Seidel S, Bilger F, Gnahs D, Hartmann J, Kuper H (2013). Informelles Lernen Erwachsener. Weiterbildungsverhalten in Deutschland. Resultate des Adult Education Survey 2012.

[CR34] Leifels, A. (2021). Weiterbildung bricht in der Krise ein – Bedarf an Digitalkompetenzen wächst. *KfW Research – Fokus Volkswirtschaft 329*. https://www.kfw.de/PDF/Download-Center/Konzernthemen/Research/PDF-Dokumente-Fokus-Volkswirtschaft/Fokus-2021/Fokus-Nr.-329-April-2021-Weiterbildung-Corona.pdf. Zugegriffen: 27. Juni 2022.

[CR35] Li, J., Valero, A., & Ventura, G. (2020). Trends in job-related training and policies for building future skills into the recovery. *CVER Discussion Paper 033*. London. https://cver.lse.ac.uk/textonly/cver/pubs/cverdp033.pdf. Zugegriffen: 22. Nov. 2021.

[CR48] NEPS-Netzwerk (2021). Nationales Bildungspanel, Scientific Use File der Startkohorte Erwachsene, *Leibniz-Institut für Bildungsverläufe (LIfBi), Bamberg*, 10.5157/NEPS:SC6:12.1.0.

[CR43] van Nieuwenhove L, de Wever B (2021). Why are low-educated adults underrepresented in adult education? Studying the role of educational background in expressing learning needs and barriers. Studies in Continuing Education.

[CR36] OECD (2021). Adult Learning and COVID-19: How much informal and non-formal learning are workers missing? *Tackling Coronavirus (Covid-19)*. https://read.oecd-ilibrary.org/view/?ref=1069_1069729-q3oh9e4dsm&title=Adult-Learning-and-COVID-19-How-much-informal-and-non-formal-learning-are-workers-missing. Zugegriffen: 7. Dez. 2021.

[CR37] Reiter S (2022). Zugewanderte Erwerbstätige in der betrieblichen Weiterbildung: Befunde aus dem AES-Migra. Zeitschrift für Weiterbildungsforschung.

[CR38] Schiener J (2006). Bildungserträge in der Erwerbsgesellschaft. Analysen zur Karrieremobilität.

[CR39] Schiener J, Wolter F, Rudolphi U, Becker R, Schulze A (2013). Weiterbildung im betrieblichen Kontext. Bildungskontexte. Strukturelle Voraussetzungen und Ursachen ungleicher Bildungschancen.

[CR40] Schmidt-Hertha B (2021). Die Pandemie als Digitalisierungsschub?. Hessische Blätter für Volksbildung.

[CR41] Seyda S (2021). Digitale Lernmedien beflügeln die betriebliche Weiterbildung: Ergebnisse der zehnten IW-Weiterbildungserhebung. IW-Trends.

[CR42] Tuijnman A, Boström A-K (2002). Changing notions of lifelong education and lifelong learning. International Review of Education.

[CR44] Wigfield A, Eccles JS (2000). Expectancy value theory of achievement motivation. Contemporary Educational Psychology.

[CR45] Wuppertaler Kreis (2020). Trends in der Weiterbildung – Verbandsumfrage 2020. https://www.wkr-ev.de/trends20/wktrends2020.pdf. Zugegriffen: 22. Nov. 2021.

[CR46] Zoch G (2022). Participation in job-related training—is there a parenthood training penalty?.

[CR47] Zoch G, Bächmann A-C, Vicari B (2021). Who cares when care closes? Care-arrangements and parental working conditions during the COVID-19 pandemic in Germany. European Societies.

