# Supplementary material for: Polarizing or reducing unequal participation opportunities? Impact of the COVID-19 pandemic on adult vocational learning
Source: Z Erziehwiss. 2023 Feb 21:1–30. [Article in German] Online ahead of print. doi: 10.1007/s11618-023-01142-2 (PMC9942025; doi:10.1007/s11618-023-01142-2)
Supplement: Supplementary file 1 [file 11618_2023_1142_MOESM1_ESM.pdf]

Online-Anhang

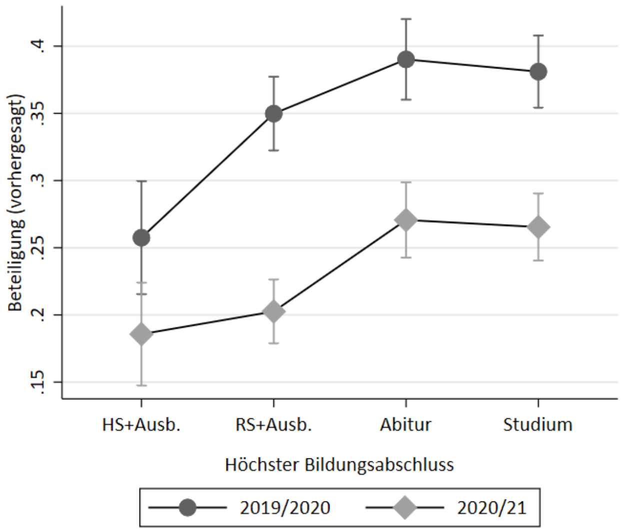

Abbildung A1. Beteiligung an nonformaler berufsbezogener Bildung nach höchstem Bildungsabschluss (vorhergesagte Werte aus Modell 4, logistische Regression)

Quelle: NEPS:SC6:12.1.0 (doi:10.5157/NEPS:SC6:12.1.0) & Konsortialdaten B146, eigene Berechnung.

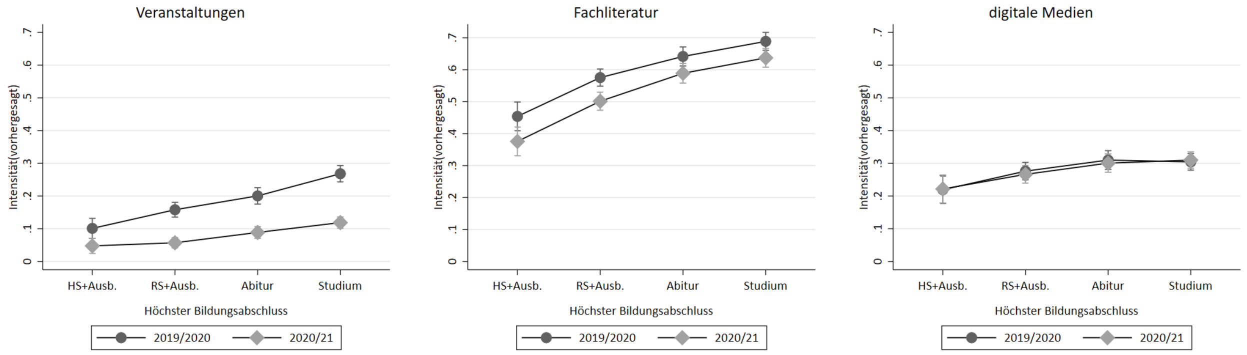

Abbildung A2. Beteiligung an informeller berufsbezogener Bildung nach höchstem Bildungsabschluss (vorhergesagte Werte aus Modell 4, logistische Regression)

Quelle: NEPS:SC6:12.1.0 (doi:10.5157/NEPS:SC6:12.1.0) & Konsortialdaten B146, eigene Berechnung.

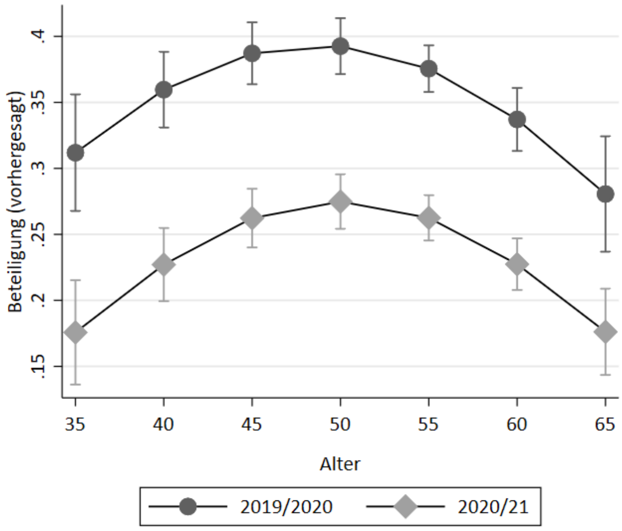

Abbildung A3. Beteiligung an nonformaler berufsbezogener Bildung nach Alter (vorhergesagte Werte aus Modell 4, logistische Regression)

Quelle: NEPS:SC6:12.1.0 (doi:10.5157/NEPS:SC6:12.1.0) & Konsortialdaten B146, eigene Berechnung.

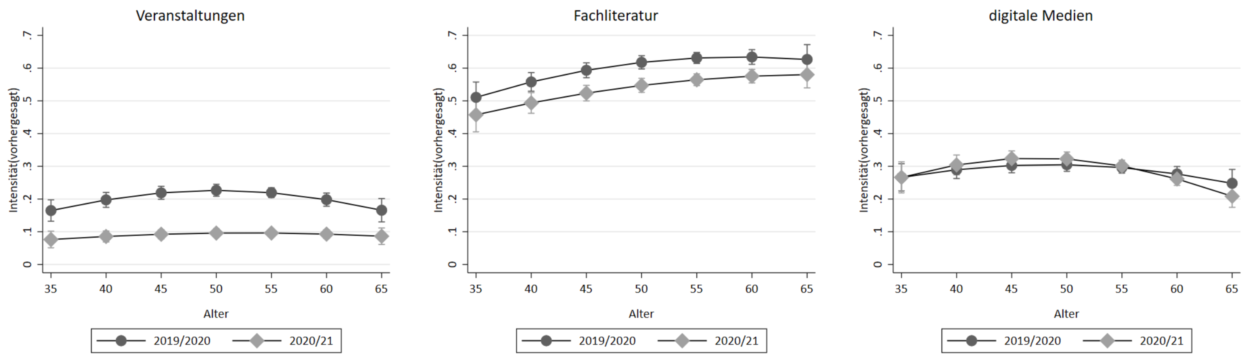

Abbildung A4. Beteiligung an informeller berufsbezogener Bildung nach Alter (vorhergesagte Werte aus Modell 4, logistische Regression)

Quelle: NEPS:SC6:12.1.0 (doi:10.5157/NEPS:SC6:12.1.0) & Konsortialdaten B146, eigene Berechnung.

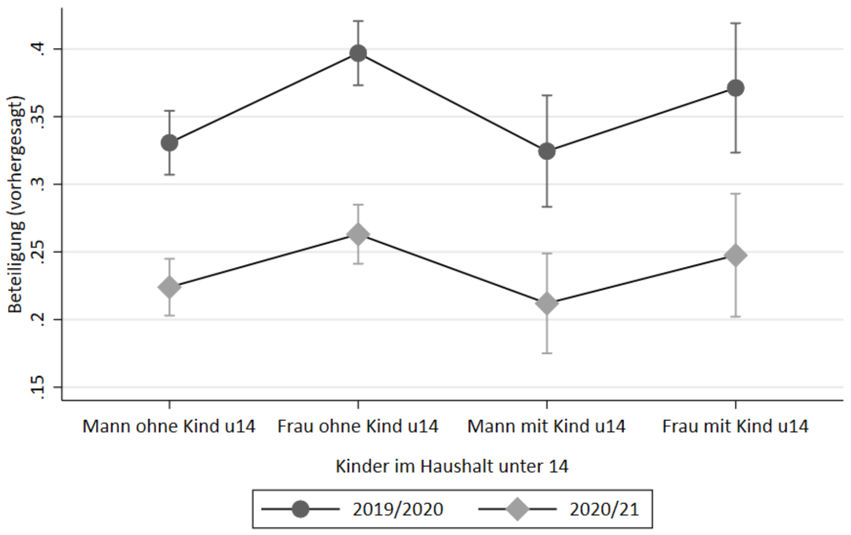

Abbildung A5. Beteiligung an nonformaler berufsbezogener Bildung nach Geschlecht und Kindern unter 14 im Haushalt (vorhergesagte Werte aus Modell 4, logistische Regression)

Quelle: NEPS:SC6:12.1.0 (doi:10.5157/NEPS:SC6:12.1.0) & Konsortialdaten B146, eigene Berechnung.

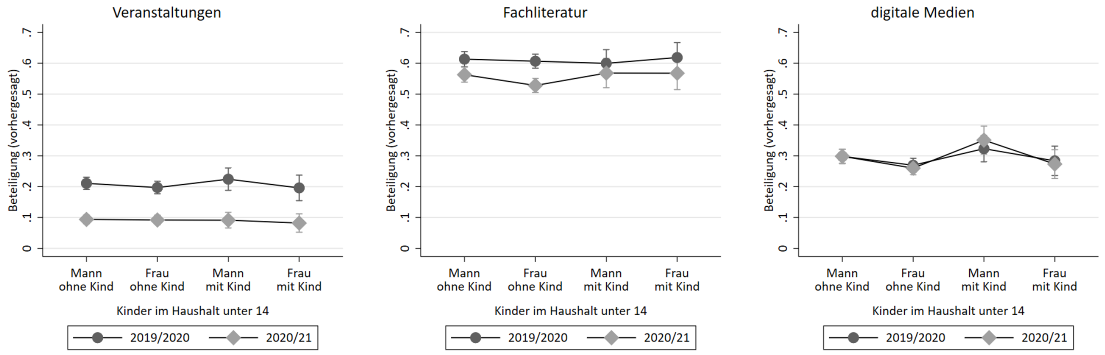

Abbildung A6. Beteiligung an informeller berufsbezogener Bildung nach Geschlecht und Kindern unter 14 im Haushalt (vorhergesagte Werte aus Modell 4, logistische Regression)

Quelle: NEPS:SC6:12.1.0 (doi:10.5157/NEPS:SC6:12.1.0) & Konsortialdaten B146, eigene Berechnung.

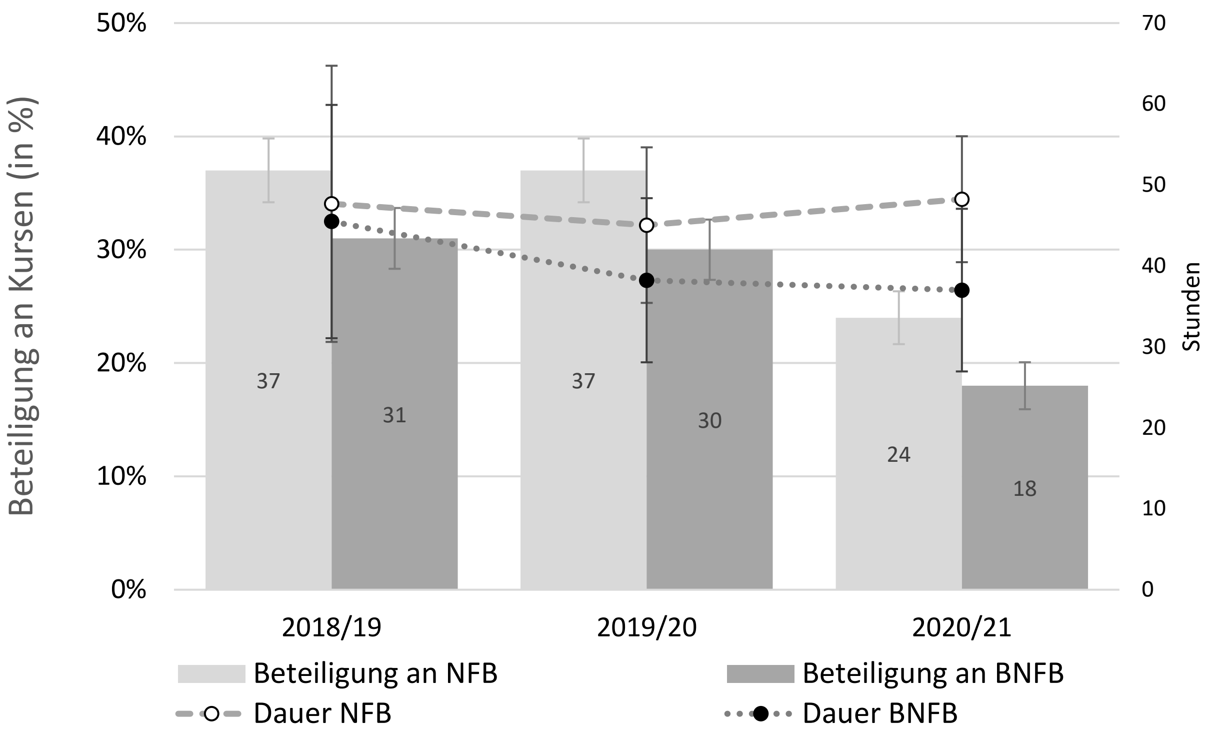

Abbildung A7. Beteiligung an nonformaler Bildung und Umfang (in % und Stunden)

Hinweis: Dargestellt ist die Beteiligung an nonformaler Bildung insgesamt (NFB) und berufsbezogener nonformaler Bildung (BNFB) in Prozent (linke Achse) sowie die Dauer in Stunden aller teilgenommenen Kurse mit und ohne Berufsbezug (rechte Achse). *Quelle*: NEPS:SC6:12.1.0 (doi:10.5157/NEPS:SC6:12.1.0) & Konsortialdaten B146, 2018-2021, gewichtet, 95-% Konfidenzintervalle, eigene Berechnung.

Tabelle A1: Verteilung der abhängigen und unabhängigen Variablen (ungewichtet)

	**Vor der Pandemie (2019/20)**				**Während der Pandemie (2020/21)**			
	MW	SD	Min	Max	MW	SD	Min	Max
Beteiligung an nonform. BWB	0.36	0.48	0.00	1.00	**0.24**	0.43	0.00	1.00
Beteiligung an inform. BWB: Veranstaltungen	0.21	0.40	0.00	1.00	**0.09**	0.29	0.00	1.00
Beteiligung an inform. BWB: Fachliteratur	0.61	0.49	0.00	1.00	**0.55**	0.50	0.00	1.00
Beteiligung an inform. BWB: digitale Medien	0.29	0.45	0.00	1.00	0.29	0.45	0.00	1.00
Migrationshintergrund	0.15	0.36	0.00	1.00	0.15	0.36	0.00	1.00
Ostdeutschland	0.19	0.39	0.00	1.00	0.19	0.39	0.00	1.00
Frau	0.49	0.50	0.00	1.00	0.49	0.50	0.00	1.00
Kinder unter 14 im Haushalt	0.35	0.72	0.00	4.00	**0.32**	0.70	0.00	4.00
Partner/in im Haushalt	0.78	0.41	0.00	1.00	0.78	0.42	0.00	1.00
Alter (in Jahren)	51.94	8.17	33.00	65.00	**52.98**	8.17	34.00	66.00
max. Hauptschule + Ausbildung	0.14	0.34	0.00	1.00	0.14	0.34	0.00	1.00
Realschule + Ausbildung	0.30	0.46	0.00	1.00	0.30	0.46	0.00	1.00
Abitur	0.22	0.41	0.00	1.00	0.22	0.41	0.00	1.00
Studium	0.35	0.48	0.00	1.00	0.35	0.48	0.00	1.00
Beruflicher Status (ISEI/10)	5.30	2.16	0.00	8.90	5.30	2.16	0.00	8.90
Arbeitszeit (in 10 Std./Woche)	3.51	1.03	1.00	6.00	**3.34**	1.22	0.00	6.00
Jobdauer (in 10 Jahren)	1.22	1.05	0.01	4.65	**1.28**	1.06	0.01	4.73
Selbständig/mithelfend/freie Mitarbeit	0.12	0.32	0.00	1.00	0.12	0.32	0.00	1.00
Homeoffice: kein Zugang	0.71	0.46	0.00	1.00	**0.50**	0.50	0.00	1.00
Homeoffice: Zugang	0.22	0.41	0.00	1.00	**0.42**	0.49	0.00	1.00
Homeoffice: keine Angabe	0.08	0.27	0.00	1.00	0.08	0.27	0.00	1.00
Nicht-systemrelevanter Beruf	0.47	0.50	0.00	1.00	0.47	0.50	0.00	1.00
Systemrelevanter Beruf	0.51	0.50	0.00	1.00	0.51	0.50	0.00	1.00
Keine Angabe zum Beruf	0.02	0.14	0.00	1.00	0.02	0.14	0.00	1.00
Kurzarbeit/Freistellung	0.00	0.00	0.00	0.00	**0.16**	0.37	0.00	1.00
Betriebl. Weiterbildungsstruktur (Index)	2.21	1.58	0.00	4.00	2.19	1.60	0.00	4.00
Land-/Forstwirtschaft	0.01	0.11	0.00	1.00	0.01	0.11	0.00	1.00
Bergbau/Steine/Erden	0.00	0.05	0.00	1.00	0.00	0.05	0.00	1.00
Verarbeitendes Gewerbe	0.21	0.41	0.00	1.00	0.21	0.41	0.00	1.00
Ver- und Entsorgung	0.02	0.13	0.00	1.00	0.02	0.13	0.00	1.00
Baugewerbe	0.03	0.17	0.00	1.00	0.03	0.17	0.00	1.00
Handel/Kraftfahrzeuge	0.06	0.24	0.00	1.00	0.06	0.24	0.00	1.00
Verkehr/Lagerei	0.03	0.17	0.00	1.00	0.03	0.17	0.00	1.00
Gastgewerbe	0.01	0.12	0.00	1.00	0.01	0.12	0.00	1.00
Information/Kommunikation	0.04	0.21	0.00	1.00	0.04	0.21	0.00	1.00
Finanz-/Versicherungs-DL	0.05	0.22	0.00	1.00	0.05	0.22	0.00	1.00
Grundstücks-/Wohnungswesen	0.01	0.07	0.00	1.00	0.01	0.07	0.00	1.00
freiberufl./wiss./techn. DL	0.05	0.22	0.00	1.00	0.05	0.22	0.00	1.00
sonst. wirtschaftl. DL	0.02	0.14	0.00	1.00	0.02	0.14	0.00	1.00
öffentliche Verwaltung	0.10	0.30	0.00	1.00	0.10	0.30	0.00	1.00
Erziehung/Unterricht	0.09	0.28	0.00	1.00	0.09	0.28	0.00	1.00
Gesundheits-/Sozialwesen	0.15	0.35	0.00	1.00	0.15	0.35	0.00	1.00
Kunst/Unterhaltung/Erholung	0.01	0.12	0.00	1.00	0.01	0.12	0.00	1.00
Nicht-Regierungsorganisationen	0.02	0.15	0.00	1.00	0.02	0.15	0.00	1.00
Sonstige Dienstleistungen	0.03	0.18	0.00	1.00	0.03	0.18	0.00	1.00
unklar	0.04	0.20	0.00	1.00	0.04	0.20	0.00	1.00

Hervorhebung kennzeichnet statistisch signifikante Mittelwertunterschiede zwischen den beiden Wellen.

Quelle: NEPS:SC6:12.1.0 (doi:10.5157/NEPS:SC6:12.1.0) & Konsortialdaten B146, ungewichtete Daten, *N*=4.203.

Tabelle A2: Verteilung der abhängigen und unabhängigen Variablen (gewichtet)

	**Vor der Pandemie (2019/20)**				**Während der Pandemie (2020/21)**			
	MW	SD	Min	Max	MW	SD	Min	Max
Beteiligung an nonform. BWB	0.30	0.46	0.00	1.00	0.18	0.38	0.00	1.00
Beteiligung an inform. BWB: Veranstaltungen	0.14	0.35	0.00	1.00	0.07	0.25	0.00	1.00
Beteiligung an inform. BWB: Fachliteratur	0.50	0.50	0.00	1.00	0.45	0.50	0.00	1.00
Beteiligung an inform. BWB: digitale Medien	0.26	0.44	0.00	1.00	0.26	0.44	0.00	1.00
Migrationshintergrund	0.27	0.44	0.00	1.00	0.27	0.44	0.00	1.00
Ostdeutschland	0.19	0.39	0.00	1.00	0.19	0.39	0.00	1.00
Frau	0.47	0.50	0.00	1.00	0.47	0.50	0.00	1.00
Kinder unter 14 im Haushalt	0.44	0.79	0.00	4.00	0.41	0.76	0.00	4.00
Partner/in im Haushalt	0.74	0.44	0.00	1.00	0.73	0.44	0.00	1.00
Alter (in Jahren)	49.00	8.81	33.00	65.00	50.06	8.79	34.00	66.00
max. Hauptschule + Ausbildung	0.25	0.44	0.00	1.00	0.25	0.44	0.00	1.00
Realschule + Ausbildung	0.37	0.48	0.00	1.00	0.37	0.48	0.00	1.00
Abitur	0.24	0.43	0.00	1.00	0.24	0.43	0.00	1.00
Studium	0.13	0.34	0.00	1.00	0.13	0.34	0.00	1.00
Beruflicher Status (ISEI/10)	4.56	2.08	0.00	8.90	4.56	2.08	0.00	8.90
Arbeitszeit (in 10 Std./Woche)	3.48	1.05	1.00	6.00	3.33	1.20	0.00	6.00
Jobdauer (in 10 Jahren)	1.11	1.01	0.01	4.65	1.16	1.03	0.01	4.73
Selbständig/mithelfend/freie Mitarbeit	0.11	0.31	0.00	1.00	0.11	0.31	0.00	1.00
Homeoffice: kein Zugang	0.74	0.44	0.00	1.00	0.59	0.49	0.00	1.00
Homeoffice: Zugang	0.18	0.38	0.00	1.00	0.33	0.47	0.00	1.00
Homeoffice: keine Angabe	0.08	0.27	0.00	1.00	0.08	0.28	0.00	1.00
Nicht-systemrelevanter Beruf	0.50	0.50	0.00	1.00	0.50	0.50	0.00	1.00
Systemrelevanter Beruf	0.48	0.50	0.00	1.00	0.48	0.50	0.00	1.00
Keine Angabe zum Beruf	0.02	0.15	0.00	1.00	0.02	0.15	0.00	1.00
Kurzarbeit/Freistellung	0.00	0.00	0.00	0.00	0.18	0.39	0.00	1.00
Betriebl. Weiterbildungsstruktur (Index)	2.02	1.62	0.00	4.00	1.97	1.59	0.00	4.00
Land-/Forstwirtschaft	0.01	0.09	0.00	1.00	0.01	0.09	0.00	1.00
Bergbau/Steine/Erden	0.00	0.07	0.00	1.00	0.00	0.07	0.00	1.00
Verarbeitendes Gewerbe	0.23	0.42	0.00	1.00	0.23	0.42	0.00	1.00
Ver- und Entsorgung	0.01	0.12	0.00	1.00	0.01	0.12	0.00	1.00
Baugewerbe	0.04	0.20	0.00	1.00	0.04	0.20	0.00	1.00
Handel/Kraftfahrzeuge	0.08	0.28	0.00	1.00	0.08	0.28	0.00	1.00
Verkehr/Lagerei	0.04	0.20	0.00	1.00	0.04	0.20	0.00	1.00
Gastgewerbe	0.02	0.15	0.00	1.00	0.02	0.15	0.00	1.00
Information/Kommunikation	0.04	0.20	0.00	1.00	0.04	0.20	0.00	1.00
Finanz-/Versicherungs-DL	0.05	0.21	0.00	1.00	0.05	0.21	0.00	1.00
Grundstücks-/Wohnungswesen	0.01	0.08	0.00	1.00	0.01	0.08	0.00	1.00
freiberufl./wiss./techn. DL	0.04	0.20	0.00	1.00	0.04	0.20	0.00	1.00
sonst. wirtschaftl. DL	0.02	0.13	0.00	1.00	0.02	0.13	0.00	1.00
öffentliche Verwaltung	0.10	0.29	0.00	1.00	0.10	0.29	0.00	1.00
Erziehung/Unterricht	0.05	0.22	0.00	1.00	0.05	0.22	0.00	1.00
Gesundheits-/Sozialwesen	0.13	0.34	0.00	1.00	0.13	0.34	0.00	1.00
Kunst/Unterhaltung/Erholung	0.02	0.14	0.00	1.00	0.02	0.14	0.00	1.00
Nicht-Regierungsorganisationen	0.01	0.12	0.00	1.00	0.01	0.12	0.00	1.00
Sonstige Dienstleistungen	0.04	0.19	0.00	1.00	0.04	0.19	0.00	1.00
unklar	0.05	0.21	0.00	1.00	0.05	0.21	0.00	1.00

Quelle: NEPS:SC6:12.1.0 (doi:10.5157/NEPS:SC6:12.1.0) & Konsortialdaten B146, gewichtete Daten, *N*=4.203.

Tabelle A3: Veränderung der Beteiligung an berufsbezogener Weiterbildung während der Covid-19 Pandemie (LPM & Logit, gepooled)

	**nonformale BWB**			**informelle BWB: Veranstaltungen**			**informelle BWB: Fachliteratur**			**informelle BWB: digitale Medien**		
	(1)	(2)	(3)	(1)	(2)	(3)	(1)	(2)	(3)	(1)	(2)	(3)
Welle 2020/21 (ref. 2019/20)	-0.12***	-0.12***	-0.12***	-0.12***	-0.12***	-0.12***	-0.06***	-0.07***	-0.07***	-0.00	-0.01	-0.01
	(0.01)	(0.01)	(0.01)	(0.01)	(0.01)	(0.01)	(0.01)	(0.01)	(0.01)	(0.01)	(0.01)	(0.01)
Migrationshintergrund *(d)*		0.03*	0.03*		-0.02	-0.02*		-0.00	-0.00		0.02	0.02
		(0.01)	(0.01)		(0.01)	(0.01)		(0.01)	(0.01)		(0.01)	(0.01)
Ostdeutschland *(d)*		0.02	0.03*		-0.02	-0.01		-0.03**	-0.04**		0.00	0.01
		(0.01)	(0.01)		(0.01)	(0.01)		(0.01)	(0.01)		(0.01)	(0.01)
Frau *(d)*		0.05***	0.03**		-0.01	-0.03**		-0.02	-0.05***		-0.04***	-0.05***
		(0.01)	(0.01)		(0.01)	(0.01)		(0.01)	(0.01)		(0.01)	(0.01)
Kinder <14 im Haushalt		-0.00	-0.00		-0.01	-0.01		-0.01	-0.01		0.00	0.00
		(0.01)	(0.01)		(0.01)	(0.01)		(0.01)	(0.01)		(0.01)	(0.01)
Partner im Haushalt *(d)*		-0.01	-0.01		0.00	0.01		-0.01	-0.00		-0.00	0.00
		(0.01)	(0.01)		(0.01)	(0.01)		(0.01)	(0.01)		(0.01)	(0.01)
Alter		0.04***	0.04***		0.02**	0.02**		0.02**	0.02***		0.03***	0.03***
		(0.01)	(0.01)		(0.01)	(0.01)		(0.01)	(0.01)		(0.01)	(0.01)
Alter, quadriert		-0.00***	-0.00***		-0.00**	-0.00**		-0.00*	-0.00**		-0.00***	-0.00***
		(0.00)	(0.00)		(0.00)	(0.00)		(0.00)	(0.00)		(0.00)	(0.00)
Realschule + Ausbildung		0.04**	0.04*		0.03**	0.03**		0.12***	0.12***		0.05**	0.04**
		(0.01)	(0.01)		(0.01)	(0.01)		(0.02)	(0.02)		(0.02)	(0.02)
Abitur		0.10***	0.09***		0.07***	0.07***		0.20***	0.20***		0.08***	0.07***
		(0.02)	(0.02)		(0.01)	(0.01)		(0.02)	(0.02)		(0.02)	(0.02)
Studium		0.09***	0.08***		0.12***	0.12***		0.25***	0.23***		0.09***	0.07***
		(0.02)	(0.02)		(0.01)	(0.01)		(0.02)	(0.02)		(0.02)	(0.02)
beruflicher Status (ISEI/10)		0.01***	0.01**		0.02***	0.02***		0.04***	0.04***		0.03***	0.02***
		(0.00)	(0.00)		(0.00)	(0.00)		(0.00)	(0.00)		(0.00)	(0.00)
Arbeitszeit, in 10 Wo.std.		0.02***	0.02***		0.02***	0.02***		0.01*	0.02***		0.01	0.01*
		(0.00)	(0.00)		(0.00)	(0.00)		(0.01)	(0.01)		(0.00)	(0.00)
Jobdauer, in 10 Jahren		-0.02***	-0.02***		-0.02***	-0.02***		-0.02***	-0.01**		-0.02**	-0.02**
		(0.00)	(0.00)		(0.00)	(0.00)		(0.01)	(0.01)		(0.01)	(0.01)
Selbständig/freie MA *(d)*		0.04*	0.03		0.10***	0.07***		0.26***	0.23***		0.19***	0.18***
		(0.02)	(0.02)		(0.02)	(0.02)		(0.01)	(0.02)		(0.02)	(0.02)
Homeoffice: Zugang		0.03*	0.03*		0.03***	0.04***		0.04***	0.05***		0.09***	0.08***
		(0.01)	(0.01)		(0.01)	(0.01)		(0.01)	(0.01)		(0.01)	(0.01)

Tabelle A3 *Fortsetzung*

	nonformale BWB			Veranstaltungen			Fachliteratur			digitale Medien		
	(1)	(2)	(3)	(1)	(2)	(3)	(1)	(2)	(3)	(1)	(2)	(3)
Homeoffice: keine Angabe		0.01	0.01		-0.03*	-0.03*		-0.00	0.01		0.02	0.01
		(0.02)	(0.02)		(0.01)	(0.01)		(0.02)	(0.02)		(0.02)	(0.02)
systemrelevanter Beruf *(d)*		0.06***	0.04***		-0.01	-0.02**		0.04***	0.01		0.02	0.01
		(0.01)	(0.01)		(0.01)	(0.01)		(0.01)	(0.01)		(0.01)	(0.01)
systemrelevanter Beruf: k.A.		0.03	0.01		0.10*	0.08*		0.15***	0.13***		0.08*	0.09*
		(0.03)	(0.03)		(0.04)	(0.04)		(0.03)	(0.03)		(0.04)	(0.04)
Kurzarbeit/Freistellung *(d)*		-0.02	0.00		-0.01	-0.01		-0.00	0.01		-0.03	-0.02
		(0.02)	(0.02)		(0.02)	(0.02)		(0.02)	(0.02)		(0.02)	(0.02)
betriebl. Weiterbildungsstruktur *(d)*		0.05***	0.05***		0.00	0.00		0.02***	0.02***		0.03***	0.03***
		(0.00)	(0.00)		(0.00)	(0.00)		(0.00)	(0.00)		(0.00)	(0.00)
Land-/Forstwirtschaft			0.06			0.07			0.26***			0.03
			(0.04)			(0.04)			(0.04)			(0.05)
Bergbau/Steine/Erden			-0.14			-0.09*			-0.11			0.19
			(0.08)			(0.04)			(0.09)			(0.10)
Ver- und Entsorgung			0.04			-0.01			0.02			0.02
			(0.04)			(0.03)			(0.04)			(0.04)
Baugewerbe			0.03			-0.03			0.05			0.00
			(0.03)			(0.02)			(0.03)			(0.03)
Handel/Kraftfahrzeuge			0.02			-0.01			0.02			0.03
			(0.02)			(0.02)			(0.02)			(0.02)
Verkehr/Lagerei			0.04			-0.08***			-0.00			0.00
			(0.03)			(0.02)			(0.03)			(0.03)
Gastgewerbe			-0.04			0.00			0.12**			0.16***
			(0.03)			(0.04)			(0.04)			(0.05)
Information/Kommunikation			0.04			0.00			0.03			0.10***
			(0.03)			(0.02)			(0.03)			(0.03)
Finanz-/Versicherungs-DL			0.07**			-0.01			-0.04			0.15***
			(0.03)			(0.02)			(0.03)			(0.03)
Grundstücks-/Wohnungswesen			0.11			0.03			0.20**			0.00
			(0.06)			(0.05)			(0.06)			(0.06)
freiberufl./wiss./techn. DL			0.10***			0.03			0.08**			0.06*
			(0.03)			(0.02)			(0.03)			(0.02)
sonst. wirtschaftl. DL			0.05			-0.04			0.11**			0.10*
			(0.03)			(0.03)			(0.04)			(0.04)

Tabelle A3 *Fortsetzung*

	nonformale WB			Veranstaltungen			Fachliteratur			digitale Medien		
	(1)	(2)	(3)	(1)	(2)	(3)	(1)	(2)	(3)	(1)	(2)	(3)
Öffentliche Verwaltung			0.04			0.01			0.04*			-0.04*
			(0.02)			(0.01)			(0.02)			(0.02)
Erziehung/Unterricht			0.14***			0.01			0.16***			0.13***
			(0.02)			(0.01)			(0.02)			(0.02)
Gesundheits-/Sozialwesen			0.10***			0.10***			0.18***			0.06***
			(0.02)			(0.02)			(0.02)			(0.02)
Kunst/Unterhaltung/Erholung			-0.00			0.02			0.24***			0.14**
			(0.04)			(0.03)			(0.04)			(0.05)
NGOs			0.09*			0.00			0.11***			0.00
			(0.03)			(0.02)			(0.03)			(0.03)
Sonstige DL			0.02			0.04			0.05			-0.01
			(0.03)			(0.02)			(0.03)			(0.03)
unklar			0.06*			0.02			0.05*			0.04
			(0.02)			(0.02)			(0.03)			(0.02)
p	0.00	0.00	0.00	0.00	0.00	0.00	0.00	0.00	0.00	0.90	0.00	0.00
R^2 (angepasst)	0.02	0.09	0.10									
Log Likelihood				-3426.35	-3090.58	-3043.36	-5704.14	-5041.36	-4938.19	-5044.80	-4673.35	-4600.33
AIC				6856.69	6225.15	6168.73	11412.28	10126.71	9958.37	10093.60	9390.71	9282.66
BIC				6870.77	6379.96	6457.23	11426.36	10281.52	10246.88	10107.67	9545.52	9571.16

Standardfehler in Klammern, *d* für Dummy-Variablen. * p < 0.05, ** p < 0.01, *** p < 0.001. *N*=4.203.

Quelle: NEPS:SC6:12.1.0 (doi:10.5157/NEPS:SC6:12.1.0) & Konsortialdaten B146, eigene Berechnung

Tabelle A4: Teilnahme an nonformaler berufsbezogener Weiterbildung (LPM, vollständige Modelle)

	(1a)	(1b)	(2a)	(2b)	(3a)	(3b)	(4a)	(4b)	(5a)	(5b)
	2019/20	2020/21	2019/20	2020/21	2019/20	2020/21	2019/20	2020/21	2019/20	2020/21
Migrationshintergrund *(d)*	0.03	0.01	0.04	0.01	0.04	0.01	0.05*	0.01	0.05*	0.01
	(0.02)	(0.02)	(0.02)	(0.02)	(0.02)	(0.02)	(0.02)	(0.02)	(0.02)	(0.02)
Ostdeutschland *(d)*	0.01	0.03	0.01	0.03	0.01	0.03	0.02	0.03	0.01	0.03*
	(0.02)	(0.02)	(0.02)	(0.02)	(0.02)	(0.02)	(0.02)	(0.02)	(0.02)	(0.02)
Frau *(d)*	0.05***	0.04**	0.08***	0.05**	0.06***	0.04**	0.06***	0.04**	0.04**	0.02
	(0.01)	(0.01)	(0.02)	(0.01)	(0.02)	(0.01)	(0.02)	(0.01)	(0.02)	(0.01)
Kinder <14 im Haushalt	0.00	0.00	0.00	0.00	0.00	-0.00	-0.00	-0.00	0.00	-0.00
	(0.01)	(0.01)	(0.01)	(0.01)	(0.01)	(0.01)	(0.01)	(0.01)	(0.01)	(0.01)
Partner im Haushalt *(d)*	-0.01	-0.01	-0.01	-0.01	-0.00	-0.01	-0.01	-0.01	-0.01	-0.01
	(0.02)	(0.02)	(0.02)	(0.02)	(0.02)	(0.02)	(0.02)	(0.02)	(0.02)	(0.02)
Alter	0.05***	0.05***	0.05***	0.05***	0.04***	0.05***	0.04***	0.04***	0.04***	0.04***
	(0.01)	(0.01)	(0.01)	(0.01)	(0.01)	(0.01)	(0.01)	(0.01)	(0.01)	(0.01)
Alter, quadriert	-0.00***	-0.00***	-0.00***	-0.00***	-0.00***	-0.00***	-0.00***	-0.00***	-0.00***	-0.00***
	(0.00)	(0.00)	(0.00)	(0.00)	(0.00)	(0.00)	(0.00)	(0.00)	(0.00)	(0.00)
Realschule + Ausbildung	0.13***	0.04*	0.09***	0.02	0.09***	0.01	0.08***	0.00	0.07**	0.00
	(0.02)	(0.02)	(0.02)	(0.02)	(0.02)	(0.02)	(0.02)	(0.02)	(0.02)	(0.02)
Abitur	0.19***	0.13***	0.14***	0.10***	0.14***	0.09***	0.12***	0.07***	0.11***	0.07**
	(0.02)	(0.02)	(0.02)	(0.02)	(0.02)	(0.02)	(0.02)	(0.02)	(0.02)	(0.02)
Studium	0.23***	0.16***	0.13***	0.09***	0.12***	0.07**	0.11***	0.07**	0.09***	0.06**
	(0.02)	(0.02)	(0.03)	(0.02)	(0.03)	(0.02)	(0.03)	(0.02)	(0.03)	(0.02)
beruflicher Status (ISEI/10)			0.03***	0.02***	0.03***	0.01***	0.02***	0.01*	0.01**	0.01
			(0.00)	(0.00)	(0.00)	(0.00)	(0.00)	(0.00)	(0.00)	(0.00)
Arbeitszeit, in 10 Wo.std.			0.03***	0.02***	0.03***	0.02***	0.02**	0.02**	0.03***	0.02**
			(0.01)	(0.01)	(0.01)	(0.01)	(0.01)	(0.01)	(0.01)	(0.01)
Jobdauer, in 10 Jahren			-0.01	-0.02**	-0.01	-0.02**	-0.02*	-0.02***	-0.02*	-0.02***
			(0.01)	(0.01)	(0.01)	(0.01)	(0.01)	(0.01)	(0.01)	(0.01)
Selbständig/freie MA *(d)*			-0.12***	-0.06***	-0.12***	-0.07***	0.04	0.03	0.04	0.02
			(0.02)	(0.02)	(0.02)	(0.02)	(0.03)	(0.02)	(0.03)	(0.02)
Homeoffice: Zugang					0.04*	0.05***	0.03	0.04*	0.03	0.04**
					(0.02)	(0.02)	(0.02)	(0.02)	(0.02)	(0.02)
Homeoffice: keine Angabe					-0.02	0.05	-0.02	0.04	-0.02	0.04
					(0.03)	(0.03)	(0.03)	(0.03)	(0.03)	(0.03)

Tabelle A4 *Fortsetzung*

	(1a) 2019/20	(1b) 2020/21	(2a) 2019/20	(2b) 2020/21	(3a) 2019/20	(3b) 2020/21	(4a) 2019/20	(4b) 2020/21	(5a) 2019/20	(5b) 2020/21
systemrelevanter Beruf					0.11***	0.05***	0.09***	0.04**	0.06***	0.03
					(0.01)	(0.01)	(0.01)	(0.01)	(0.02)	(0.01)
Keine Angabe zum Beruf					0.10	0.05	0.04	0.02	0.01	0.00
					(0.05)	(0.05)	(0.05)	(0.05)	(0.05)	(0.05)
Kurzarbeit/Freistellung					--	-0.04*	--	-0.02	--	-0.01
					--	(0.02)	--	(0.02)	--	(0.02)
Betriebl. Weiterb.struktur							0.06***	0.04***	0.06***	0.04***
							(0.01)	(0.00)	(0.01)	(0.00)
Land-/Forstwirtschaft									0.04	0.08
									(0.06)	(0.06)
Bergbau/Steine/Erden									-0.11	-0.17*
									(0.13)	(0.08)
Ver- und Entsorgung									0.06	0.01
									(0.06)	(0.05)
Baugewerbe									0.04	0.02
									(0.04)	(0.03)
Handel/Kraftfahrzeuge									0.04	0.01
									(0.03)	(0.02)
Verkehr/Lagerei									0.05	0.03
									(0.04)	(0.04)
Gastgewerbe									-0.06	-0.02
									(0.05)	(0.04)
Informat./Kommunik.									0.05	0.03
									(0.04)	(0.03)
Finanzen/Versicherungen									0.08*	0.06
									(0.04)	(0.04)
Immobilien									0.13	0.08
									(0.09)	(0.08)
freiberufl./wiss./techn. DL									0.10**	0.10**
									(0.04)	(0.04)
sonst. wirtschaftl. DL									0.04	0.06
									(0.05)	(0.04)
Öffentliche Verwaltung									0.06*	0.01
									(0.03)	(0.03)

Tabelle A4 *Fortsetzung*

	(1a) 2019/20	(1b) 2020/21	(2a) 2019/20	(2b) 2020/21	(3a) 2019/20	(3b) 2020/21	(4a) 2019/20	(4b) 2020/21	(5a) 2019/20	(5b) 2020/21
Erziehung/Unterricht									0.18^{***}	0.10^{***}
									(0.03)	(0.03)
Gesundheits-/Sozialwesen									0.10^{***}	0.09^{***}
									(0.03)	(0.03)
Kunst/Unterhalt./Erholung									0.00	-0.00
									(0.06)	(0.05)
Nicht-Regierungs-Organis.									0.16^{**}	0.01
									(0.05)	(0.05)
Sonstige Dienstleistungen									0.01	0.03
									(0.04)	(0.04)
Wirtschaftszweig unklar									0.09^{*}	0.03
									(0.04)	(0.03)
R^2 (angepasst)	0.03	0.03	0.05	0.04	0.06	0.05	0.09	0.06	0.10	0.06
p	0.00	0.00	0.00	0.00	0.00	0.00	0.00	0.00	0.00	0.00

Standardfehler in Klammern, *d* für Dummy-Variablen. * p < 0.05, ** p < 0.01, *** p < 0.001. *N*=4.203.

Quelle: NEPS:SC6:12.1.0 (doi:10.5157/NEPS:SC6:12.1.0) & Konsortialdaten B146, eigene Berechnung

Tabelle A5: Teilnahme an informeller berufsbezogener Weiterbildung: Veranstaltungen (Logit, vollständige Modelle)

	(1a)<br>2019/20	(1b)<br>2020/21	(2a)<br>2019/20	(2b)<br>2020/21	(3a)<br>2019/20	(3b)<br>2020/21	(4a)<br>2019/20	(4b)<br>2020/21	(5a)<br>2019/20	(5b)<br>2020/21
Migrationshintergrund *(d)*	-0.03*	-0.01	-0.03*	-0.01	-0.03	-0.01	-0.03	-0.01	-0.04*	-0.01
	(0.02)	(0.01)	(0.02)	(0.01)	(0.02)	(0.01)	(0.02)	(0.01)	(0.02)	(0.01)
Ostdeutschland *(d)*	-0.01	-0.02	-0.02	-0.02	-0.02	-0.02	-0.01	-0.02	-0.01	-0.02
	(0.02)	(0.01)	(0.01)	(0.01)	(0.02)	(0.01)	(0.02)	(0.01)	(0.02)	(0.01)
Frau *(d)*	-0.05***	-0.01	-0.03	-0.00	-0.02	-0.00	-0.02	-0.00	-0.04**	-0.02
	(0.01)	(0.01)	(0.01)	(0.01)	(0.01)	(0.01)	(0.01)	(0.01)	(0.01)	(0.01)
Kinder <14 im Haushalt	-0.01	-0.01	-0.01	-0.01	-0.01	-0.01	-0.01	-0.01	-0.01	-0.01
	(0.01)	(0.01)	(0.01)	(0.01)	(0.01)	(0.01)	(0.01)	(0.01)	(0.01)	(0.01)
Partner im Haushalt *(d)*	0.01	-0.00	0.01	0.00	0.01	0.00	0.01	0.00	0.01	0.00
	(0.02)	(0.01)	(0.01)	(0.01)	(0.02)	(0.01)	(0.02)	(0.01)	(0.02)	(0.01)
Alter	0.04***	0.01	0.03***	0.01	0.03**	0.01	0.03**	0.01	0.03**	0.01
	(0.01)	(0.01)	(0.01)	(0.01)	(0.01)	(0.01)	(0.01)	(0.01)	(0.01)	(0.01)
Alter, quadriert	-0.00***	-0.00	-0.00***	-0.00	-0.00**	-0.00	-0.00**	-0.00	-0.00**	-0.00
	(0.00)	(0.00)	(0.00)	(0.00)	(0.00)	(0.00)	(0.00)	(0.00)	(0.00)	(0.00)
Realschule + Ausbildung	0.06***	0.01	0.06***	0.01	0.08**	0.01	0.08**	0.01	0.08**	0.01
	(0.01)	(0.01)	(0.02)	(0.01)	(0.03)	(0.01)	(0.03)	(0.01)	(0.03)	(0.01)
Abitur	0.13***	0.06***	0.10***	0.04**	0.12***	0.04**	0.12***	0.04**	0.12***	0.04*
	(0.02)	(0.01)	(0.02)	(0.01)	(0.03)	(0.01)	(0.03)	(0.02)	(0.03)	(0.02)
Studium	0.26***	0.13***	0.19***	0.08***	0.18***	0.07***	0.18***	0.07***	0.18***	0.07***
	(0.02)	(0.01)	(0.02)	(0.02)	(0.03)	(0.02)	(0.03)	(0.02)	(0.03)	(0.02)
beruflicher Status (ISEI/10)			0.02***	0.02***	0.02***	0.01***	0.02***	0.01***	0.02***	0.01***
			(0.00)	(0.00)	(0.00)	(0.00)	(0.00)	(0.00)	(0.00)	(0.00)
Arbeitszeit, in 10 Wo.std.			0.03***	0.01***	0.03***	0.01**	0.03***	0.01**	0.03***	0.01**
			(0.01)	(0.00)	(0.01)	(0.00)	(0.01)	(0.00)	(0.01)	(0.00)
Jobdauer, in 10 Jahren			-0.01*	-0.02***	-0.01*	-0.02***	-0.01*	-0.02***	-0.01	-0.02***
			(0.01)	(0.00)	(0.01)	(0.01)	(0.01)	(0.01)	(0.01)	(0.01)
Selbständig/freie MA *(d)*			0.13***	0.06***	0.10***	0.06***	0.11***	0.07**	0.09***	0.05*
			(0.02)	(0.02)	(0.02)	(0.02)	(0.02)	(0.02)	(0.02)	(0.02)
Homeoffice: Zugang					0.04**	0.02*	0.04**	0.02*	0.05**	0.03**
					(0.01)	(0.01)	(0.01)	(0.01)	(0.01)	(0.01)
Homeoffice: keine Angabe					-0.06*	-0.01	-0.06*	-0.01	-0.06*	-0.01
					(0.03)	(0.02)	(0.03)	(0.02)	(0.03)	(0.02)

Tabelle A5 *Fortsetzung*

	(1a)<br>2019/20	(1b)<br>2020/21	(2a)<br>2019/20	(2b)<br>2020/21	(3a)<br>2019/20	(3b)<br>2020/21	(4a)<br>2019/20	(4b)<br>2020/21	(5a)<br>2019/20	(5b)<br>2020/21
systemrelevanter Beruf					-0.02*	0.00	-0.03*	0.00	-0.04**	-0.01
					(0.01)	(0.01)	(0.01)	(0.01)	(0.01)	(0.01)
Keine Angabe zum Beruf					0.13**	0.04	0.12**	0.04	0.11*	0.03
					(0.04)	(0.05)	(0.04)	(0.05)	(0.04)	(0.05)
Kurzarbeit/Freistellung					--	-0.01	--	-0.01	--	-0.01
					--	(0.01)	--	(0.01)	--	(0.01)
Betriebl. Weiterb.struktur							0.00	0.00	0.00	0.00
							(0.00)	(0.00)	(0.00)	(0.00)
Land-/Forstwirtschaft									0.08	0.06
									(0.05)	(0.05)
Bergbau/Steine/Erden									--	0.02
									--	(0.08)
Ver- und Entsorgung									0.01	-0.03
									(0.05)	(0.02)
Baugewerbe									-0.05	-0.01
									(0.04)	(0.02)
Handel/Kraftfahrzeuge									-0.06	0.04
									(0.03)	(0.03)
Verkehr/Lagerei									-0.24***	-0.01
									(0.07)	(0.02)
Gastgewerbe									-0.01	0.02
									(0.06)	(0.05)
Informat./Kommunik.									-0.01	0.02
									(0.03)	(0.02)
Finanzen/Versicherungen									-0.03	0.01
									(0.03)	(0.02)
Immobilien									0.04	0.02
									(0.07)	(0.07)
freiberufl./wiss./techn. DL									0.01	0.06**
									(0.03)	(0.02)
sonst. wirtschaftl. DL									-0.10	0.00
									(0.06)	(0.04)
Öffentliche Verwaltung									0.00	0.01
									(0.02)	(0.02)

Tabelle A5 *Fortsetzung*

	(1a) 2019/20	(1b) 2020/21	(2a) 2019/20	(2b) 2020/21	(3a) 2019/20	(3b) 2020/21	(4a) 2019/20	(4b) 2020/21	(5a) 2019/20	(5b) 2020/21
Erziehung/Unterricht									0.00	0.02
									(0.02)	(0.02)
Gesundheits-/Sozialwesen									0.09***	0.09***
									(0.02)	(0.02)
Kunst/Unterhalt./Erholung									-0.01	0.05
									(0.05)	(0.04)
Nicht-Regierungs-Organis.									0.00	0.01
									(0.04)	(0.03)
Sonstige Dienstleistungen									0.05	0.03
									(0.03)	(0.03)
Wirtschaftszweig unklar									-0.00	0.04
									(0.03)	(0.03)
Log Likelihood	-1995.78	-1207.21	-1935.25	-1166.80	-1919.52	-1161.94	-1919.26	-1161.31	-1881.62	-1140.47
AIC	4013.56	2436.42	3900.49	2363.60	3877.04	2363.88	3878.53	2364.63	3839.24	2360.94
BIC	4083.34	2506.20	3995.65	2458.75	3997.57	2490.75	4005.40	2497.84	4080.20	2614.68

Standardfehler in Klammern, *d* für Dummy-Variablen. * $p < 0.05$, ** $p < 0.01$, *** $p < 0.001$. *N*=4.203.

Quelle: NEPS:SC6:12.1.0 (doi:10.5157/NEPS:SC6:12.1.0) & Konsortialdaten B146, eigene Berechnung

Tabelle A6: Teilnahme an informeller berufsbezogener Weiterbildung: Fachliteratur (Logit, vollständige Modelle)

	(1a)<br>2019/20	(1b)<br>2020/21	(2a)<br>2019/20	(2b)<br>2020/21	(3a)<br>2019/20	(3b)<br>2020/21	(4a)<br>2019/20	(4b)<br>2020/21	(5a)<br>2019/20	(5b)<br>2020/21
Migrationshintergrund *(d)*	-0.03	0.01	-0.02	0.02	-0.02	0.02	-0.02	0.02	-0.02	0.02
	(0.02)	(0.02)	(0.02)	(0.02)	(0.02)	(0.02)	(0.02)	(0.02)	(0.02)	(0.02)
Ostdeutschland *(d)*	-0.04*	-0.05*	-0.04*	-0.04*	-0.03	-0.04	-0.03	-0.04	-0.04*	-0.04*
	(0.02)	(0.02)	(0.02)	(0.02)	(0.02)	(0.02)	(0.02)	(0.02)	(0.02)	(0.02)
Frau *(d)*	-0.02	-0.03	0.00	-0.02	-0.00	-0.03	-0.00	-0.03	-0.03*	-0.07***
	(0.01)	(0.01)	(0.02)	(0.02)	(0.02)	(0.02)	(0.02)	(0.02)	(0.02)	(0.02)
Kinder <14 im Haushalt	-0.01	0.01	-0.01	0.00	-0.02	-0.00	-0.02	-0.00	-0.02	-0.00
	(0.01)	(0.01)	(0.01)	(0.01)	(0.01)	(0.01)	(0.01)	(0.01)	(0.01)	(0.01)
Partner im Haushalt *(d)*	-0.00	-0.02	-0.00	-0.01	-0.00	-0.01	-0.01	-0.02	0.00	-0.01
	(0.02)	(0.02)	(0.02)	(0.02)	(0.02)	(0.02)	(0.02)	(0.02)	(0.02)	(0.02)
Alter	0.03**	0.02*	0.03**	0.02	0.03**	0.02	0.02*	0.02	0.03**	0.02
	(0.01)	(0.01)	(0.01)	(0.01)	(0.01)	(0.01)	(0.01)	(0.01)	(0.01)	(0.01)
Alter, quadriert	-0.00*	-0.00	-0.00*	-0.00	-0.00*	-0.00	-0.00*	-0.00	-0.00*	-0.00
	(0.00)	(0.00)	(0.00)	(0.00)	(0.00)	(0.00)	(0.00)	(0.00)	(0.00)	(0.00)
Realschule + Ausbildung	0.19***	0.17***	0.14***	0.14***	0.11***	0.13***	0.11***	0.13***	0.11***	0.12***
	(0.02)	(0.02)	(0.03)	(0.03)	(0.02)	(0.03)	(0.02)	(0.03)	(0.02)	(0.03)
Abitur	0.32***	0.32***	0.21***	0.23***	0.18***	0.22***	0.17***	0.21***	0.17***	0.21***
	(0.03)	(0.02)	(0.03)	(0.03)	(0.02)	(0.03)	(0.02)	(0.03)	(0.02)	(0.03)
Studium	0.44***	0.44***	0.27***	0.29***	0.22***	0.26***	0.22***	0.26***	0.21***	0.24***
	(0.02)	(0.02)	(0.03)	(0.03)	(0.03)	(0.03)	(0.03)	(0.03)	(0.03)	(0.03)
beruflicher Status (ISEI/10)			0.04***	0.04***	0.05***	0.04***	0.04***	0.03***	0.04***	0.03***
			(0.00)	(0.00)	(0.00)	(0.00)	(0.00)	(0.00)	(0.00)	(0.00)
Arbeitszeit, in 10 Wo.std.			0.02**	0.01	0.02**	0.01	0.02*	0.01	0.03**	0.01
			(0.01)	(0.01)	(0.01)	(0.01)	(0.01)	(0.01)	(0.01)	(0.01)
Jobdauer, in 10 Jahren			-0.01	-0.02**	-0.01	-0.02**	-0.01	-0.03***	-0.01	-0.02**
			(0.01)	(0.01)	(0.01)	(0.01)	(0.01)	(0.01)	(0.01)	(0.01)
Selbständig/freie MA *(d)*			0.24***	0.23***	0.26***	0.22***	0.32***	0.25***	0.28***	0.22***
			(0.02)	(0.02)	(0.03)	(0.02)	(0.03)	(0.02)	(0.03)	(0.02)
Homeoffice: Zugang					0.05*	0.05**	0.04*	0.05**	0.05*	0.05**
					(0.02)	(0.02)	(0.02)	(0.02)	(0.02)	(0.02)
Homeoffice: keine Angabe					0.00	-0.01	0.00	-0.02	0.00	-0.00
					(0.03)	(0.03)	(0.03)	(0.03)	(0.03)	(0.03)

Tabelle A6 *Fortsetzung*

	(1a) 2019/20	(1b) 2020/21	(2a) 2019/20	(2b) 2020/21	(3a) 2019/20	(3b) 2020/21	(4a) 2019/20	(4b) 2020/21	(5a) 2019/20	(5b) 2020/21
systemrelevanter Beruf					0.04**	0.04**	0.04*	0.04**	0.01	0.02
					(0.01)	(0.01)	(0.01)	(0.02)	(0.02)	(0.02)
Keine Angabe zum Beruf					0.22***	0.13*	0.20***	0.11*	0.17**	0.09
					(0.05)	(0.05)	(0.05)	(0.05)	(0.05)	(0.05)
Kurzarbeit/Freistellung					--	-0.01	--	-0.01	--	0.01
					--	(0.02)	--	(0.02)	--	(0.02)
Betriebl. Weiterb.struktur							0.02***	0.02**	0.02***	0.02***
							(0.01)	(0.01)	(0.01)	(0.01)
Land-/Forstwirtschaft									0.20**	0.32***
									(0.07)	(0.06)
Bergbau/Steine/Erden									-0.08	-0.12
									(0.12)	(0.12)
Ver- und Entsorgung									-0.00	0.04
									(0.05)	(0.05)
Baugewerbe									0.02	0.08
									(0.04)	(0.04)
Handel/Kraftfahrzeuge									0.01	0.04
									(0.03)	(0.03)
Verkehr/Lagerei									-0.02	0.02
									(0.04)	(0.04)
Gastgewerbe									0.11	0.13*
									(0.06)	(0.06)
Informat./Kommunik.									0.01	0.05
									(0.04)	(0.04)
Finanzen/Versicherungen									-0.05	-0.04
									(0.03)	(0.04)
Immobilien									0.16	0.23**
									(0.09)	(0.09)
freiberufl./wiss./techn. DL									0.03	0.13***
									(0.04)	(0.04)
sonst. wirtschaftl. DL									0.12*	0.11*
									(0.05)	(0.05)
Öffentliche Verwaltung									0.04	0.04
									(0.03)	(0.03)

Tabelle A6 *Fortsetzung*

	(1a) 2019/20	(1b) 2020/21	(2a) 2019/20	(2b) 2020/21	(3a) 2019/20	(3b) 2020/21	(4a) 2019/20	(4b) 2020/21	(5a) 2019/20	(5b) 2020/21
Erziehung/Unterricht									0.15***	0.18***
									(0.03)	(0.03)
Gesundheits-/Sozialwesen									0.17***	0.19***
									(0.03)	(0.03)
Kunst/Unterhalt./Erholung									0.19**	0.29***
									(0.07)	(0.06)
Nicht-Regierungs-Organis.									0.09	0.14**
									(0.05)	(0.05)
Sonstige Dienstleistungen									0.01	0.08
									(0.04)	(0.05)
Wirtschaftszweig unklar									0.03	0.07
									(0.03)	(0.04)
Log Likelihood	-2612.22	-2684.29	-2488.67	-2587.81	-2473.55	-2575.68	-2463.88	-2570.55	-2416.68	-2510.90
AIC	5246.43	5390.58	5007.35	5205.62	4985.10	5191.37	4967.76	5183.10	4911.36	5101.79
BIC	5316.21	5460.36	5102.50	5300.77	5105.63	5318.24	5094.63	5316.31	5158.76	5355.53

Standardfehler in Klammern, *d* für Dummy-Variablen. * p < 0.05, ** p < 0.01, *** p < 0.001. *N*=4.203.

Quelle: NEPS:SC6:12.1.0 (doi:10.5157/NEPS:SC6:12.1.0) & Konsortialdaten B146, eigene Berechnung

Tabelle A7: Teilnahme an informeller berufsbezogener Weiterbildung: digitale Medien (Logit, vollständige Modelle)

	(1a)<br>2019/20	(1b)<br>2020/21	(2a)<br>2019/20	(2b)<br>2020/21	(3a)<br>2019/20	(3b)<br>2020/21	(4a)<br>2019/20	(4b)<br>2020/21	(5a)<br>2019/20	(5b)<br>2020/21
Migrationshintergrund *(d)*	-0.01	0.03	-0.00	0.03	0.00	0.04	0.00	0.04	0.00	0.03
	(0.02)	(0.02)	(0.02)	(0.02)	(0.02)	(0.02)	(0.02)	(0.02)	(0.02)	(0.02)
Ostdeutschland *(d)*	-0.02	0.00	-0.01	0.01	-0.01	0.02	-0.01	0.02	-0.00	0.02
	(0.02)	(0.02)	(0.02)	(0.02)	(0.02)	(0.02)	(0.02)	(0.02)	(0.02)	(0.02)
Frau *(d)*	-0.05**	-0.05***	-0.03*	-0.04**	-0.03*	-0.05**	-0.03*	-0.05***	-0.04*	-0.06***
	(0.01)	(0.01)	(0.02)	(0.01)	(0.02)	(0.01)	(0.02)	(0.01)	(0.02)	(0.01)
Kinder <14 im Haushalt	0.01	0.01	0.00	0.01	0.00	0.00	-0.00	0.00	-0.00	0.00
	(0.01)	(0.01)	(0.01)	(0.01)	(0.01)	(0.01)	(0.01)	(0.01)	(0.01)	(0.01)
Partner im Haushalt *(d)*	0.03	-0.02	0.04*	-0.02	0.03	-0.03	0.03	-0.03	0.03	-0.02
	(0.02)	(0.02)	(0.02)	(0.02)	(0.02)	(0.02)	(0.02)	(0.02)	(0.02)	(0.02)
Alter	0.02*	0.04***	0.02*	0.04***	0.02*	0.04***	0.02*	0.04***	0.02	0.04***
	(0.01)	(0.01)	(0.01)	(0.01)	(0.01)	(0.01)	(0.01)	(0.01)	(0.01)	(0.01)
Alter, quadriert	-0.00*	-0.00***	-0.00*	-0.00***	-0.00*	-0.00***	-0.00*	-0.00***	-0.00	-0.00***
	(0.00)	(0.00)	(0.00)	(0.00)	(0.00)	(0.00)	(0.00)	(0.00)	(0.00)	(0.00)
Realschule + Ausbildung	0.09***	0.08***	0.07**	0.06*	0.07**	0.05*	0.06*	0.04	0.06*	0.04
	(0.02)	(0.02)	(0.02)	(0.02)	(0.03)	(0.02)	(0.03)	(0.02)	(0.03)	(0.02)
Abitur	0.16***	0.16***	0.11***	0.11***	0.11***	0.09***	0.10***	0.08**	0.08**	0.06*
	(0.02)	(0.02)	(0.02)	(0.02)	(0.03)	(0.03)	(0.03)	(0.03)	(0.03)	(0.03)
Studium	0.23***	0.25***	0.11***	0.13***	0.10**	0.09**	0.09**	0.09**	0.08**	0.07*
	(0.02)	(0.02)	(0.03)	(0.03)	(0.03)	(0.03)	(0.03)	(0.03)	(0.03)	(0.03)
beruflicher Status (ISEI/10)			0.03***	0.03***	0.03***	0.03***	0.03***	0.03***	0.03***	0.02***
			(0.00)	(0.00)	(0.00)	(0.00)	(0.00)	(0.00)	(0.00)	(0.00)
Arbeitszeit, in 10 Wo.std.			0.02*	0.01	0.02**	0.00	0.02*	-0.00	0.02*	0.00
			(0.01)	(0.01)	(0.01)	(0.01)	(0.01)	(0.01)	(0.01)	(0.01)
Jobdauer, in 10 Jahren			-0.02*	-0.01	-0.02*	-0.01	-0.02**	-0.01	-0.02**	-0.01
			(0.01)	(0.01)	(0.01)	(0.01)	(0.01)	(0.01)	(0.01)	(0.01)
Selbständig/freie MA *(d)*			0.09***	0.15***	0.06**	0.13***	0.15***	0.22***	0.13***	0.21***
			(0.02)	(0.02)	(0.02)	(0.02)	(0.03)	(0.03)	(0.03)	(0.03)
Homeoffice: Zugang					0.09***	0.10***	0.08***	0.10***	0.07***	0.08***
					(0.02)	(0.02)	(0.02)	(0.02)	(0.02)	(0.02)
Homeoffice: keine Angabe					0.03	0.01	0.03	0.00	0.02	-0.01
					(0.03)	(0.03)	(0.03)	(0.03)	(0.03)	(0.03)

Tabelle A7 *Fortsetzung*

	(1a) 2019/20	(1b) 2020/21	(2a) 2019/20	(2b) 2020/21	(3a) 2019/20	(3b) 2020/21	(4a) 2019/20	(4b) 2020/21	(5a) 2019/20	(5b) 2020/21
systemrelevanter Beruf					0.03	0.02	0.02	0.01	0.01	0.00
					(0.01)	(0.01)	(0.01)	(0.01)	(0.02)	(0.01)
Keine Angabe zum Beruf					0.12*	0.09	0.09	0.07	0.09	0.08
					(0.05)	(0.06)	(0.05)	(0.06)	(0.05)	(0.06)
Kurzarbeit/Freistellung					--	-0.03	--	-0.02	--	-0.02
					--	(0.02)	--	(0.02)	--	(0.02)
Betriebl. Weiterb.struktur							0.03***	0.03***	0.03***	0.03***
							(0.01)	(0.01)	(0.01)	(0.01)
Land-/Forstwirtschaft									0.06	0.01
									(0.06)	(0.06)
Bergbau/Steine/Erden									0.18	0.18
									(0.12)	(0.15)
Ver- und Entsorgung									-0.03	0.07
									(0.05)	(0.05)
Baugewerbe									-0.04	0.04
									(0.05)	(0.04)
Handel/Kraftfahrzeuge									0.00	0.06
									(0.03)	(0.03)
Verkehr/Lagerei									0.02	-0.02
									(0.04)	(0.04)
Gastgewerbe									0.13*	0.17**
									(0.06)	(0.06)
Informat./Kommunik.									0.10**	0.10**
									(0.03)	(0.03)
Finanzen/Versicherungen									0.12***	0.17***
									(0.03)	(0.04)
Immobilien									0.02	-0.01
									(0.10)	(0.09)
freiberufl./wiss./techn. DL									0.07*	0.04
									(0.03)	(0.03)
sonst. wirtschaftl. DL									0.10	0.09
									(0.05)	(0.06)
Öffentliche Verwaltung									-0.06	-0.04
									(0.03)	(0.02)

Tabelle A7 *Fortsetzung*

	(1a) 2019/20	(1b) 2020/21	(2a) 2019/20	(2b) 2020/21	(3a) 2019/20	(3b) 2020/21	(4a) 2019/20	(4b) 2020/21	(5a) 2019/20	(5b) 2020/21
Erziehung/Unterricht									0.10***	0.17***
									(0.03)	(0.03)
Gesundheits-/Sozialwesen									0.06*	0.06*
									(0.03)	(0.03)
Kunst/Unterhalt./Erholung									0.08	0.21**
									(0.06)	(0.07)
Nicht-Regierungs-Organis.									-0.03	0.03
									(0.05)	(0.05)
Sonstige Dienstleistungen									-0.04	0.01
									(0.04)	(0.04)
Wirtschaftszweig unklar									0.02	0.05
									(0.04)	(0.03)
Log Likelihood	-2443.57	-2408.64	-2391.57	-2348.05	-2374.63	-2320.96	-2358.20	-2305.77	-2325.15	-2259.08
AIC	4909.15	4839.28	4813.13	4726.11	4787.26	4681.91	4756.40	4653.54	4728.30	4598.15
BIC	4978.93	4909.06	4908.28	4821.26	4907.78	4808.79	4883.27	4786.75	4975.70	4851.89

Standardfehler in Klammern, *d* für Dummy-Variablen. * p < 0.05, ** p < 0.01, *** p < 0.001. *N*=4.203.

Quelle: NEPS:SC6:12.1.0 (doi:10.5157/NEPS:SC6:12.1.0) & Konsortialdaten B146, eigene Berechnung
